# Microsecond MD Simulation and Multiple-Conformation Virtual Screening to Identify Potential Anti-COVID-19 Inhibitors Against SARS-CoV-2 Main Protease

**DOI:** 10.3389/fchem.2020.595273

**Published:** 2021-01-13

**Authors:** Chandrabose Selvaraj, Umesh Panwar, Dhurvas Chandrasekaran Dinesh, Evzen Boura, Poonam Singh, Vikash Kumar Dubey, Sanjeev Kumar Singh

**Affiliations:** ^1^Computer Aided Drug Design and Molecular Modeling Lab, Department of Bioinformatics, Alagappa University, Karaikudi, India; ^2^Section of Molecular Biology and Biochemistry, Institute of Organic Chemistry and Biochemistry AS CR, v.v.i., Prague, Czechia; ^3^Corrosion and Materials Protection Division, Council of Scientific and Industrial Research (CSIR)-Central Electrochemical Research Institute, Karaikudi, India; ^4^School of Biochemical Engineering, Indian Institute of Technology (BHU), Varanasi, India

**Keywords:** SARS-CoV-2 main protease, COVID-19, TCM, natural products, molecular dynamics, ensemble sampling

## Abstract

The recent pandemic outbreak of COVID-19, caused by severe acute respiratory syndrome coronavirus 2 (SARS-CoV-2), raised global health and economic concerns. Phylogenetically, SARS-CoV-2 is closely related to SARS-CoV, and both encode the enzyme main protease (M^pro^/3CL^pro^), which can be a potential target inhibiting viral replication. Through this work, we have compiled the structural aspects of M^pro^ conformational changes, with molecular modeling and 1-μs MD simulations. Long-scale MD simulation resolves the mechanism role of crucial amino acids involved in protein stability, followed by ensemble docking which provides potential compounds from the Traditional Chinese Medicine (TCM) database. These lead compounds directly interact with active site residues (His41, Gly143, and Cys145) of M^pro^, which plays a crucial role in the enzymatic activity. Through the binding mode analysis in the S1, S1′, S2, and S4 binding subsites, screened compounds may be functional for the distortion of the oxyanion hole in the reaction mechanism, and it may lead to the inhibition of M^pro^ in SARS-CoV-2. The hit compounds are naturally occurring compounds; they provide a sustainable and readily available option for medical treatment in humans infected by SARS-CoV-2. Henceforth, extensive analysis through molecular modeling approaches explained that the proposed molecules might be promising SARS-CoV-2 inhibitors for the inhibition of COVID-19, subjected to experimental validation.

## Introduction

The novel coronavirus “Severe Acute Respiratory Syndrome Coronavirus-2” (SARS-CoV-2) became a disease of interest with the initial alert of several pneumonia cases on 31 December 2019 by China (Lai et al., 2020). Since then, the virus has spread globally, representing a major threat to public health, and eventually, on 11 March 2020, WHO announced it as a pandemic (Vannabouathong et al., 2020). As of 19^th^ November 2020, there are over 55.6 million cases globally, with a 2.6% case-mortality rate (https://ourworldindata.org/mortality-risk-covid). From the coronavirus family, SARS-CoV-2 is the seventh virus that infects humans, and like other viruses in that family, like SARS-CoV and MERS-CoV, SARS-CoV-2 has a high lethality (Li et al., 2020; Zhu et al., 2020). COVID-19 can be either asymptomatic or symptomatic, and severe cases result in pneumonia, multiple-organ failure, and eventual death (Ayres, 2020). The SARS-CoV-2 belongs to the family *Coronaviridae* and subfamily *Coronavirinae*, which contains enveloped, positive-sense single-stranded RNA (+ssRNA) viruses whose external glycoprotein spikes from the envelope appear as a “*corona*,” which is Latin for crown or halo-like, hence the virus family name (Pal et al., 2020). The SARS-CoV-2 whole-genome sequence data (29,903 nucleotides) shows an overall 82% sequence identity with SARS-CoV. Sequence analyses confirm SARS-CoV-2's possible natural reservoirs are horseshoe bats (*Rhinolophus sp*.), though the intermediate host is still not clear (Zhao et al., 2020; Zheng, 2020). Available metagenomics data and sequence similarities of CoV from animals suggest that Malayan pangolins (*Manis javanica*, long-snouted, ant-eating mammals) could be a possible intermediate host (Rabi et al., 2020; Wahba et al., 2020). These lethal ssRNA viruses are highly flexible to adapt, acquiring new mutations which enable them to move into a new host and to elude available conventional drugs and making vaccine development challenging (Hanney et al., 2020). For temporary solutions, repurposing FDA-approved drugs, especially low-molecular-weight drugs, and using available cutting-edge techniques like CRISPR/Cas13d targeting SARS-CoV-2 viral RNA can also be rapid approaches to fight COVID-19 (Gao et al., 2020; Mohammadi et al., 2020; Singh et al., 2020). In this pandemic, it is mandatory to analyze the drug targets of SARS-CoV-2 with available FDA-approved compounds and also to find new inhibitors against those targets (Gil et al., 2020; Meyer-Almes, 2020; Saul and Einav, 2020). Therefore, structural biology and computational virtual screening of antiviral molecules that target key viral proteins can be comparatively faster and more effective than developing vaccines or therapeutic antibodies to fight against SARS-CoV-2 (Battisti et al., 2020; Frances-Monerris et al., 2020; Shyr et al., 2020). The host dependency factors mediating virus infection will be the key to understanding effective molecular targets for developing broadly acting antiviral therapeutics against SARS-CoV-2 (Tharappel et al., 2020).

**Figure 1:**
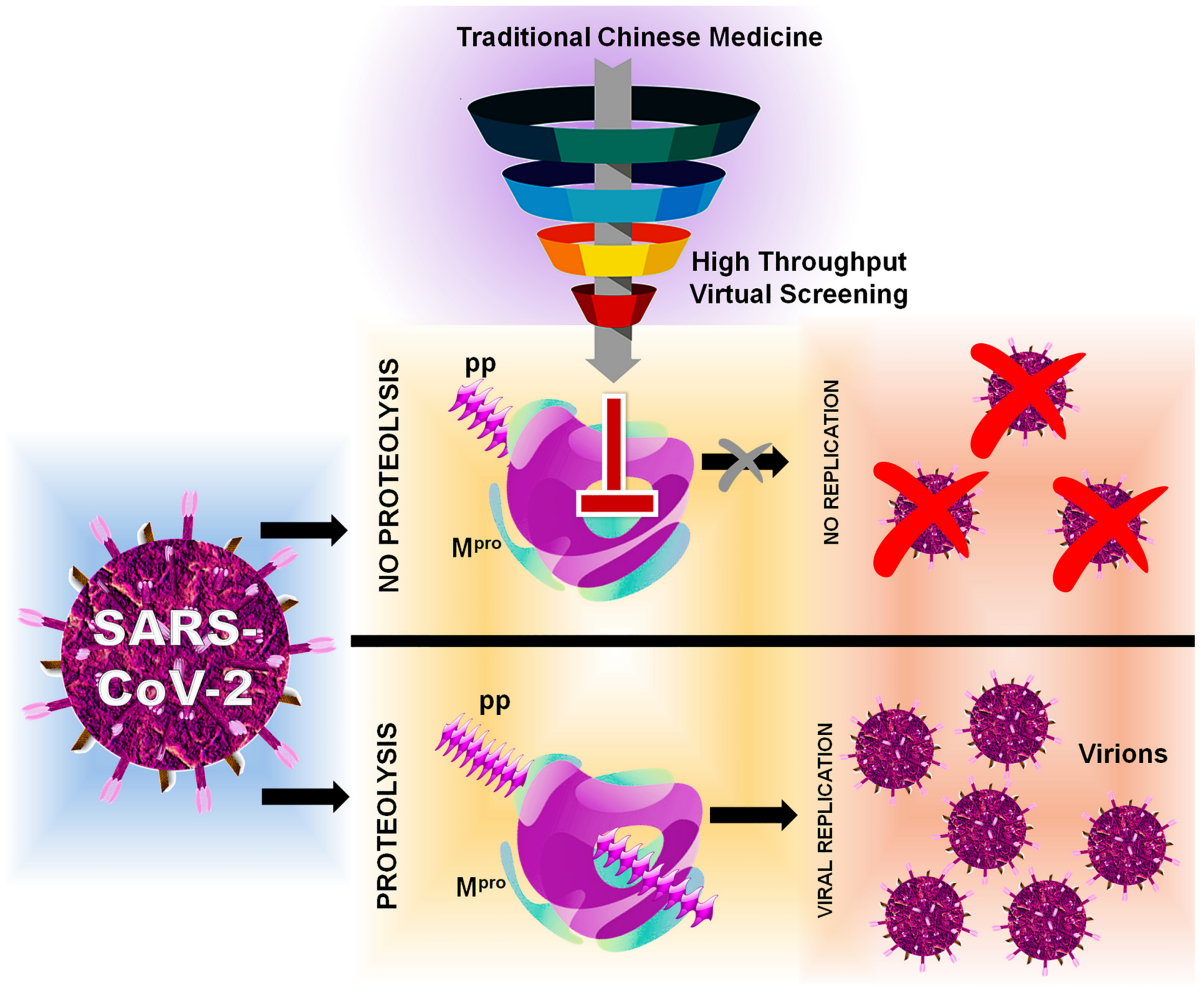
Flowchart of HTVS with SARS-CoV-2 with protein M^pro^/3CL^pro^ polyprotein processing and inhibition.

In this, SARS-CoV-2 encodes two different proteases that are crucial for viral replication, polyprotein processing, and immune regulation, namely, 3-chymotrypsin-like protease or main protease (3CL^pro^/M^pro^) and papain-like protease (PL^pro^) (Pillaiyar et al., 2016; Jeong et al., 2020; Luan et al., 2020). These two enzymes process the large polyprotein (pp) or replicase 1a and 1ab, which are translated from the viral RNA. M^pro^/3CL^pro^ has eleven cleavage sites in pp1a and pp1ab, with similar processing pathways to other coronaviruses, generating many of the non-structural proteins which are important in viral replication (Fang et al., 2008). These proteases possess a characteristic active-site architecture: glutamine in the P1 position of the substrate and (small)-X-(L/F/M)-Q↓(G/A/S)-X as a cleavage pattern (X-any amino acid). No known human protease functions with similar specificity, making M^pro^/3CL^pro^ unique and also avoid side effects or toxicity (Hilgenfeld, 2014; Eleftheriou et al., 2020). M^pro^ contains a catalytic Cys…His dyad which serves as a functionally active dimer, making it unique from the other enteroviral 3C proteases (Ullrich and Nitsche, 2020). Therefore, M^pro^ can be one of the key drug targets because of its strikingly high sequence and 3D structural similarity with M^pro^ from several other coronaviruses, for which several recently experimentally solved crystal structures for SARS-CoV-2 apo and holo forms are available in Protein Data Bank (PDB) (Gahlawat et al., 2020; Goyal and Goyal, 2020). Based on the initial structural analyses, it is already clear that drug-binding pockets of these enzymes are highly conserved as per the genome sequence analyses. Considering the functional importance, several researchers have put a mass effort via *in silico* and *in vitro* approaches, for the FDA-approved compounds (Frances-Monerris et al., 2020; Touret et al., 2020). Currently, big pharmaceutical companies worldwide are taking advantage of the available traditional Chinese medicine (TCM) databases for performing virtual screening and developing novel lead compounds for a wide range of diseases (Chen, 2011). Therefore, we have also adapted computer-aided drug discovery (CADD) approaches to screen and identify possible novel small-molecule drug-like inhibitors against SARS-CoV-2 M^pro^/3CL^pro^ available from the TCM Database@Taiwan (http://tcm.cmu.edu.tw) which include over 20,000 compounds isolated from TCMs shown in Figure 1 indicating the inhibition mechanism elucidated in this study.

## Materials and Methods

### Protein and Ligand Preparation

For the molecular modeling calculations, the crystal structure SARS-CoV-2 main protease (M^pro^/3CL^pro^) with the PDB ID: 6LU7 is prepared utilizing the standard protocol of the protein preparation wizard (Jin et al., 2020). The co-crystal ligand N3 is covalently bound with an active site amino acid Cys145, and in preparation we manually break the covalent bond and fill the open valance (Culletta et al., 2020; Wang, 2020). Here, the missing atoms and partial charges (charge alteration shown in Supplementary Figures 1a,b) are added, and the missing side-chain atoms and bond orders are refined (Sastry et al., 2013). Absolute side-chain angles of amino acids (Asn, Gln, and His) are obtained by flip, which can influence the formation of H-bonds, generating tautomers/ionized states. The intramolecular H-bonds were optimized and minimized by using the OPLS-3e force field (FF) till the RMSD threshold reaches 0.30 Å for all atoms (Selvaraj et al., 2020a). Similarly, the ligands from the TCM (Traditional Chinese Medicine) database@Taiwan (http://tcm.cmu.edu.tw/) is prepared using the LigPrep module using the OPLS-3e FF. TCM database holds extensive sources of medicinal benefits with long history, and we believe that TCM can provide the potent leads for SARS-CoV-2 M^pro^ inhibition. Ligand ionization states at pH 7.0 ± 2.0 and stereoisomers are generated for each ligand structure. Conformations up to 10 poses are generated based on the available rotatable bonds and subject to molecular modeling calculations (Sastry et al., 2013).

### Molecular Dynamics Simulation: Stage I

The prepared structure of SARS-CoV-2 M^pro^/3CL^pro^ with the presence and absence of peptidomimetic inhibitor N3 is subject to molecular dynamic (MD) simulations using the GROningen MAchine for Chemical Simulations (GROMACS 5.1.4: http://www.gromacs.org/) (Van Der Spoel et al., 2005; Selvaraj et al., 2018). This stage 1 MD simulation is performed for the timescale of 1 microsecond for understanding the structural variance occurring between the apo and ligand-bound complex. The apo and complex are subject to simple point charge (SPC) water molecules within a cubic period box of 1.0 nm distance, fixed in position between the protein and cubic box system is prepared with GROMOS96 54a7 FF (Pronk et al., 2013). The molecular topology of the N3 inhibitor is generated externally using the PRODRG web server (http://prodrg1.dyndns.org/) and then merged with protein topology files prepared by the GROMACS (Van Aalten et al., 1996; Zeng et al., 2015). The compiled system is neutralized by adding an accurate concentration of (Na^+^/Cl^−^) ions and subject to energy minimization, to remove initial steric clashes using 1,000 steps of the steepest descent algorithm via a tolerance of 10 kJ/mol/nm. Literature evidence suggests adaptation of coronaviruses to host species with different body temperatures. SARS-CoV-2 M^pro^ is a viral protein, which can adopt the human body temperature of 300–310 K, and thus we have chosen the default Berendsen thermostat with 300 K as reference temperature and Parrinello-Rahman pressure-coupling with a 1.0-bar reference pressure. The LINCS algorithm is applied as length constraints of covalent bonds, and particle-mesh Ewald is applied for computing the long-range electrostatic interactions (Chinnasamy et al., 2020a,b). The vdW and Coulomb energy cutoff values are set to 1.0 nm, and the time step is defined as 2 fs recorded in the intervals of 10 ps (Umesh et al., 2020). The minimized systems are well-equilibrated for 1,000 ps at 300 K and 1 bar pressure in NVT and NPT ensembles. Furthermore, the MD simulation is processed for the stability and time-dependent behavior of both apo and ligand-bound complexes for the long run simulation time of 1 μs (Childers and Daggett, 2018). Final trajectories are analyzed using UCSF Chimera, Visual Molecular Dynamics (VMD), GROMACS tools, and the secondary structure and ligand interactions are predicted through PDBsum.

### Preparation of Multiple Conformation-Based GRIDs

For the molecular docking, multiple grids are prepared from different conformations obtained from the 0-ns to 1-μs MD simulations. From the MD trajectories, the measure of similarity/dissimilarity is checked for conformational changes. From this, we have evidently observed the backbone stability in SARS-CoV-2 M^pro^ for the long-scale MD simulations (Evangelista Falcon et al., 2019). Thus, the conformations obtained from each 50-ns interval snapshots from 0 ns to 1 μs along with an average conformation (21 conformations) are extracted and subject to Glide Grid generation. Even though the snapshot interval of 50 ns is a bit high, the long-scale 1-μs MD simulation shows that the backbone is stable and side residues are contributing to the conformational changes (Amaro et al., 2018; Salmaso and Moro, 2018). Thus, the 50-ns interval conformations from 0-ns to 1-μs MD simulations are extracted and subjected to the grid generation method, by matching the ligand-bound pose, and the grid for glide docking is prepared (Lorber and Shoichet, 1998; Halgren et al., 2004). The box positioned on the interacting amino acids Phe140, Gly143, Cys145, His163, His164, Glu166, Gln189, and Thr190 coordinates is constructed to perform a docking analysis by including the positional region focused around 2 Å in the grid generation (Friesner et al., 2006). This process is repeatedly performed for all the 21 conformations obtained through the MD simulation.

### Virtual Screening From the TCM Database

Using the multiple-grid input in the virtual screening workflow and the prepared TCM database, high-throughput virtual screening is carried out. Within this framework, the top 10% of ligands linked to each conformation are processed from HTVS (high-throughput virtual screening) to SP docking (standard precision) and then to XP docking (extra precision) (Seifert et al., 2007). Final compounds on XP docking are processed with molecular mechanics with the generalized born surface area (MM/GBSA) method for gauging the effectiveness of interactions between a docked protein-ligand complex (Zoete et al., 2010; Selvaraj et al., 2020a). Here the average binding free energy (ΔG_bind_) is calculated based on the equation (ΔG_bind_ = ΔE_MM_ + ΔG_Solv_ + ΔG_SA_), in which ΔE_MM_ denotes minimized energies of protein and ligand, ΔG_Solv_ represents solvation-free energy, and ΔG_SA_ is the surface area energy (Tripathi et al., 2012). Best compounds from XP docking and MM/GBSA are filtered with the criteria of < −8 and < −30 kcal/mol of docking score and ΔG_bind_, respectively. Compounds surpassing the abovementioned criteria are again redocked with average conformation of the 1-μs MD simulations, using the induced fit docking (IFD) method. The IFD approach provided the selections to fix both the ligand and protein as flexible, and this furnishes the final possible best pose to describe (Selvaraj et al., 2020b). The scaling factor is defined as 0.5 for softening the potentials of protein and ligand, and the final complex pose up to twenty poses is saved. The IFD scores, which accounts for both the protein–ligand interaction energy and the total energy of the system, were calculated (Mizutani et al., 2006). For the hit compounds, theoretical validation is performed using the enrichment studies from the decoy set of the known 1,000 active sets, available in the Schrödinger database (Gani et al., 2013). This is performed for evaluating the power of hit compounds, accuracy ranking of hit compounds, and model reliability (Kalyaanamoorthy and Chen, 2014; Perez-Regidor et al., 2016).

### Molecular Dynamics Simulation: Stage II

The final best pose from the IFD docking is subject to MD simulations of the complex for the timescale of 100 ns for understanding the dynamic behavior of hit compounds with SARS-CoV-2 M^pro^ (Umesh et al., 2020). The ligand complex MD simulations are also performed as per protocol provided in the stage 1 MD simulations, and for the analysis, the root mean square deviation (RMSD), solvent-accessible surface area (SASA), and ligand-binding energy using MM-PBSA are calculated (Klimovich and Mobley, 2015; Selvaraj et al., 2018). The molecular mechanics' potential energy along with the free energy of solvation of individual complexes is analyzed using the equation (ΔG_binding_ = E_gas_ + G_sol_ – TΔS) by considering all the frames from 100 ns of MD simulation trajectories. For this calculation, the “bondi” was considered as type of radius (-rad), with an assigned default value 1, the inner dielectric constant value is quoted as 2, and for solvent, the value is assigned as 80 (Aldeghi et al., 2017). The detailed methodology of the MM/PBSA analysis is provided in the Supplementary Information.

## Results

### Main Protease Structure and Substrate-Binding Site

The architecture of SARS-CoV-2 M^pro^/3CL^pro^ comprises three domains I (1–101 residues), II (102–184 residues), and domain III (201–301 residues), which is required for enzymatic activity (Bzowka et al., 2020). The co-crystal ligand (N3 inhibitor) bound inside the active site located in between the two anti-parallel β-barrel domains (domain I and domain II), and this domain contains the catalytic Cys-His dyad, which is mechanistically crucial for cutting the polyprotein precursors. Domain III is a five-helix bundle and is primarily responsible for the dimerization and linked with domain II by a long loop (185–200 AAs). We have inspected the interactions of ligands, available with recently solved crystal structures of M^pro^ (PDB ID: 6XMK, 6Y2G, 6Y2F, 6XFN, 6ZRT, 6ZRU, 7BQY, 7BRP, 7BUY, 6LZE, and 6M0K), and we noticed that all the co-crystal ligands have a similar binding mode (details provided in Supplementary Table 1). Among the structures, the structures reported by Dai et al. (2020) provide the inhibitors 11a and 11b, which strongly show the activity in the cell culture, but 11a tends to have a strong pharmacokinetic property and comes out as a strong drug candidate, by showing interactions with core important residues (Dai et al., 2020). The co-crystal ligand (PRD_002214)-based FDA repurpose screening with docking and consensus ranking provides a similar binding mode of ritonavir with a SARS-CoV-2 M^pro^ active site (Cavasotto and Di Filippo, 2020). Likewise, Wang (2020) has reported that the FDA-approved compounds, namely, carfilzomib, eravacycline, valrubicin, lopinavir, and elbasvir, have the features to bind inside the SARS-CoV-2 M^pro^ active site (Wang, 2020). Ferraz et al. (2020) have reported three FDA-approved compounds, namely, bedaquiline, glibenclamide, and miconazole, through ligand and structure-based methods. They additionally stated that conformational changes occur in the S2-binding pocket, which must be considered for the drug design approach (Ferraz et al., 2020). The active site architecture includes four subsites (S1′, S1, S2, and S4) which can accommodate the substrate recognition sequence (N-terminal—P4-P3-P2-P1↓P1′-P2′-P3′–C-terminal) and the cleavage between P1 and P1′ residues resulting in proteolytic cleavage, thereby processing the viral polyproteins. The Gln residue in the P1 position plays a pivotal role in substrate recognition (S1 subsite) and highly specific only for the main protease of coronavirus and 3C protease of enterovirus. The active site includes the catalytic dyad (Cys145 and His41) and several other key residues (His163, His172, and Glu166) involved in opening gates to the active site (Zhang et al., 2020). The S2 and S4 subsites form deep and shallow hydrophobic pockets, respectively, accommodating residues in P2 and P4 positions with varied specificities. The residue in the P3 position is mostly solvent exposed, and because of the absence of the S3 subsite, it can tolerate any amino acid residue. The amides from amino acids Gly143, Cys145, and Ser144 form the cysteine protease's canonical “oxyanion hole”. Recently, a report on another antiviral drug showed carmofur to inhibit the viral replication with the EC_50_ value of 24.30 μM, by binding with Gly143 and Cys145. Here carmofur shows to be bound covalently with Cys145 and the ligand fatty acid tail region readily occupies the hydrophobic S2 subsite (Jin et al., 2020).

### Molecular Dynamics Simulation: Stage I

**Figure 2:**
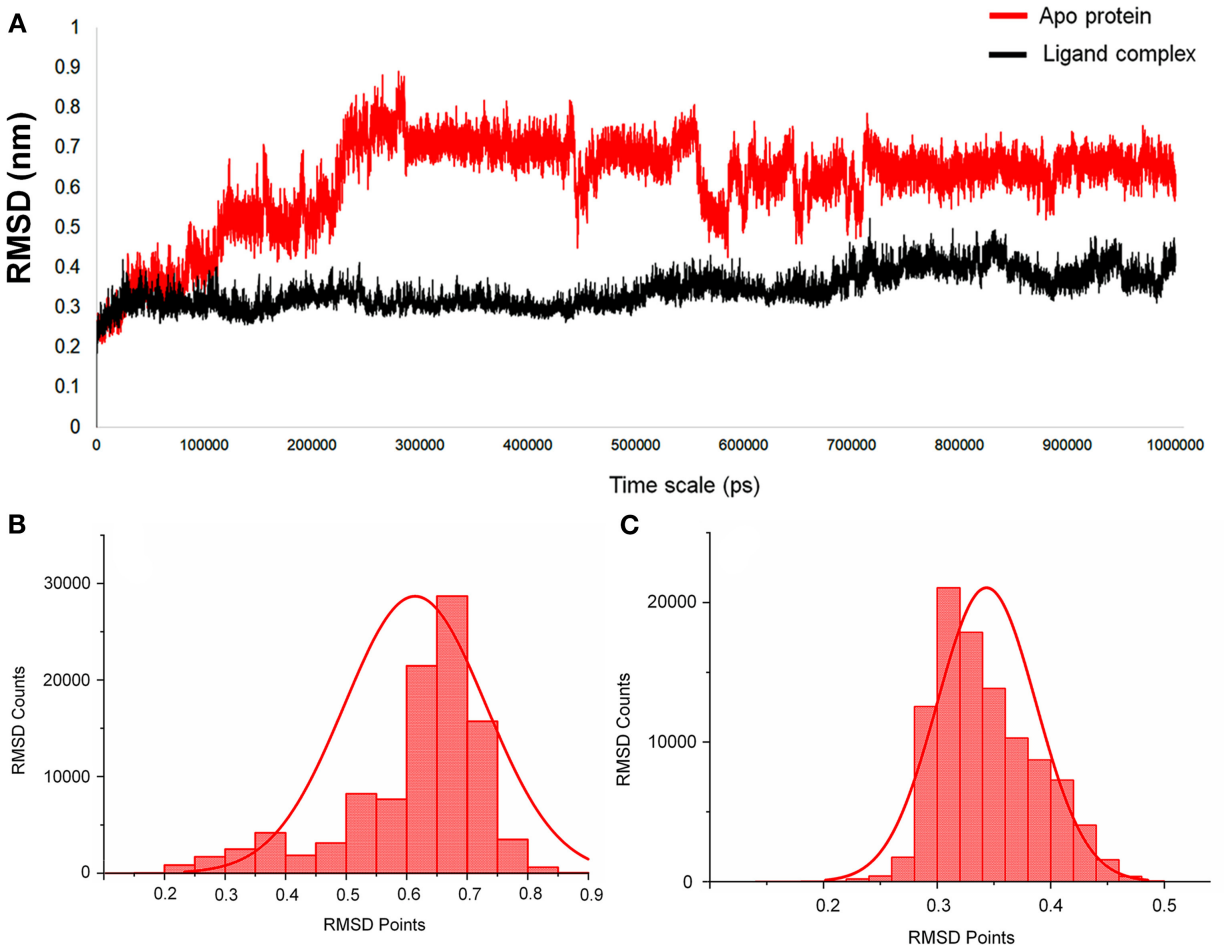
**(A)** The RMSD graph for the entire timescale of the 1-microsecond (μs) molecular dynamics (MD) simulation shown for the apo protein (red) and the protein–ligand complex (black) of SARS-CoV-2 main protease (M^pro^/3CL^pro^) co-crystal structure (PDB ID:6LU7), for exploring the conformational landscapes. **(B,C)** Histogram representing the deviation points that occur in 1-microsecond (μs) molecular dynamics (MD) simulation, and here **(B)** represents the apo SARS-CoV-2 M^pro^, while **(C)** represents the SARS-CoV-2 M^pro^ in complex with an inhibitor N3, PRD_002214 from 6LU7 co-crystal structure.

For this M^pro^ structure in both apo and holo forms, MD simulations are performed for the long-scale simulation time of 1 microsecond (1 μs). The holo form bound with the peptidomimetic N3 inhibitor (PRD_002214) remains stable throughout the MD simulations for 1 μs as shown in Figure 2A (black), and their RMSD value ranges between ~0.30 and ~0.35 nm. The absence of the peptidomimetic N3 inhibitor in the apo form shows high deviations and fluctuations in 1-μs MD simulations. The initial 100 ns shows matching with holo forms, and from the 110^th^ ns, the changes are seen in the loop regions of the apo form. At the 230^th^ ns, the RMSD values reached up to ~0.8 nm and high fluctuations are seen in the 440^th^ ns to the 700^th^ ns. The apo form stabilizes at the 720^th^ ns, and the RMSD values range between ~0.6 and ~0.8 nm until the end of the 1-μs MD simulation as shown in Figure 2A (red). The values of RMSD for both apo and holo forms are plotted in histograms provided in Figures 2B,C. For the apo form (Figure 2B), the major count of the RMSD lies from 0.6 to 0.75 nm in between the range count of 2,000 and 3,000 times. However, for the holo form (Figure 2C), the major count of the RMSD lies from 0.28 to 0.36 nm in between the range count of 1,000 to 2,000 times approximately. This histogram result along with an RMSD graph shows that the apo state is highly flexible than the holo form of SARS-CoV-2 M^pro^. For understanding the causative fluctuations with residue-wise participation in MD simulations, the root mean square fluctuation (RMSF) is calculated, and the values are plotted in Supplementary Figure 2. In addition, the active site residue values are plotted with 2D interactions in Supplementary Figure 3 and the secondary structure is provided in Supplementary Figure 4. The secondary structure along with the RMSF plot shows the high fluctuations of residues in loop regions of the apo form, while those residues show few fluctuations in holo forms. The data suggest that the amino acids Phe140, Gly143, Cys145, His163, His164, Glu166, Gln189, and Thr190 are responsible for the stable RMSD of the holo form.

In addition, the statistical values are calculated to show the impact of ligand binding in SARS-CoV-2 M^pro^ and the values are provided in Table 1. The statistical data shows the mean difference of 0.27 and median difference of 0.31 between the apo and holo forms. Separating the higher half from the lower half of the data sample shows the median values of 0.64 and 0.33 for the apo and holo forms, respectively. The standard deviation (SD) of the holo form shows 0.04, while the apo form is 0.11, demonstrating the ligand-binding effect with the difference of 0.07. This statistical data narrates that the RMSD values in apo forms are widespread, and the holo forms show to be linear and stable and tend to be close to the mean of the set. In terms of variance, the apo form with high fluctuations leads to a spread-out with value 0.01, while the holo form shows fewer fluctuations leading to a spread close to value 0.001. The statistical variance difference of 0.011 shows that the apo form is more flexible and the ligand bound in M^pro^ tends to arrest the flexibility in the 1-μs MD simulations. The range of apo lies between 0 (minimum) and 0.89 (maximum), and in this, 68% of the data lies in the interval between 0.49 and 0.73 and about 95% of the data lie in the interval between 0.37 and 0.84. However, for the holo form, the range lies between 0 (minimum) and 0.52 (maximum), about 68% of the data lies in the interval between 0.29 and 0.38, and about 95% of the data lie in the interval between 0.25 and 0.43. Since mode = 3 median−2 mean and from Table 1, the mean and median are approximately equal for apo protein, and so the distribution for apo protein can be assumed to be approximately symmetrical. Similarly, the value distributions of holo forms are symmetrical but show more stability than the apo form. The coefficient of variation for apo form and holo form are 0.19 and 0.12, respectively, which shows that the ligand-bound form is consistent and the apo state is flexible.

**Table 1:** Statistical analysis of RMSD values obtained from the 1 μs MD simulation for apo and holo forms of SARS-CoV-2 M^pro^.

**Content**	**Mean**	**Median**	**SD**	**Variance**	**Coefficient of variation**	**Min**	**Max**
Apo protein (PDB ID: 6LU7)	0.61	0.64	0.11	0.01	0.19	0	0.89
Holo protein N3-bound complex	0.34	0.33	0.04	0.00	0.12	0	0.52

### Ensemble Docking With Multiple Conformations of the Protein Structure

**Figure 3:**
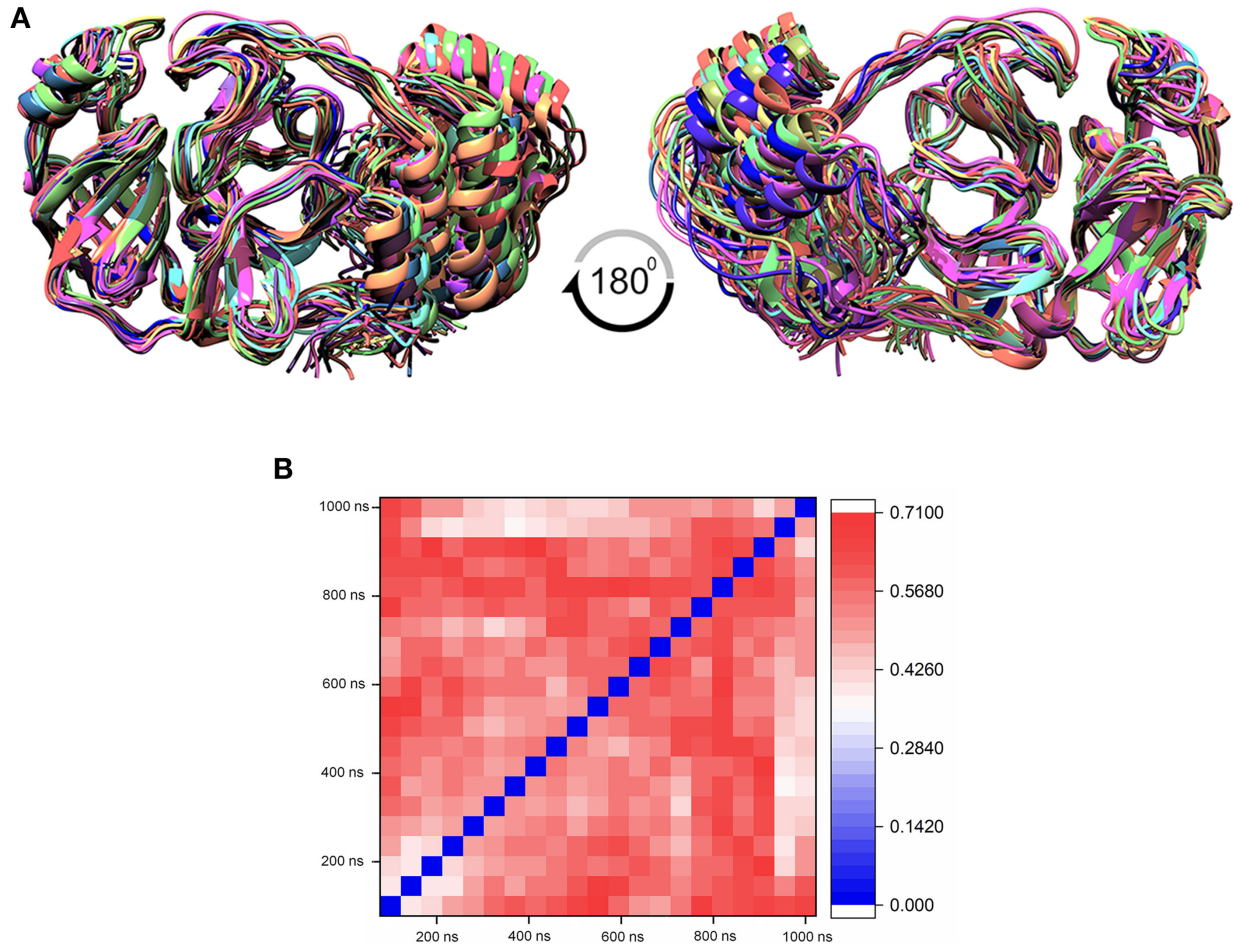
**(A)** Aligned structure of the SARS-CoV-2 main protease (M^pro^/3CL^pro^) obtained from various conformations of the MD simulation for the timescale of 1 microsecond (μs), and these multiple conformations are used for multiple-grid-based virtual screening. **(B)** Heatmap matrix for RMSD variation poses obtained from each 50-ns interval for the 1-μs MD simulation for SARS-CoV-2 M^pro^.

**Figure 4:**
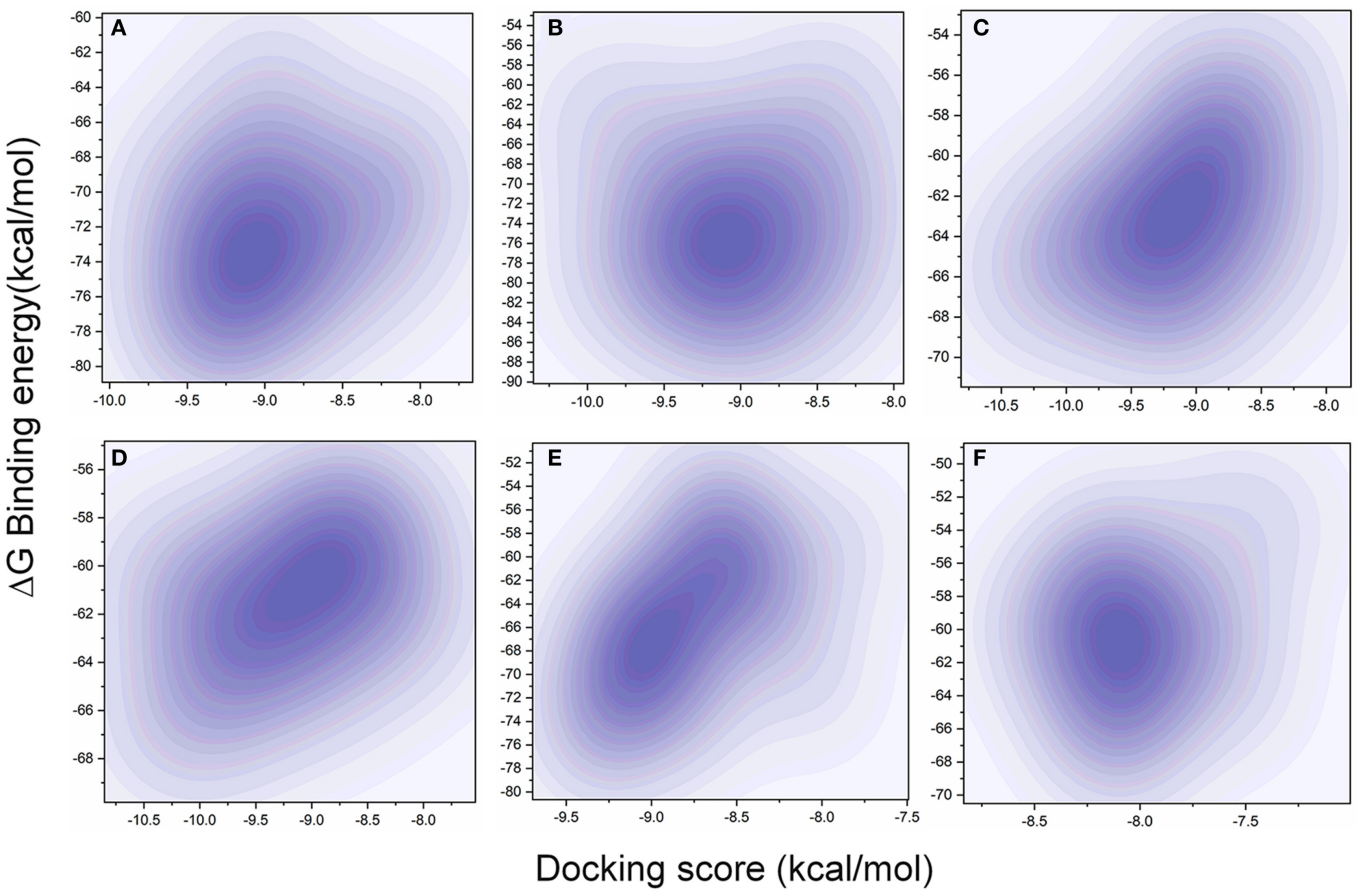
Comparison of XP docking vs binding energy (MM/GBSA) for the 20-pose multiple-protein conformation obtained from the 1-microsecond (μs) MD simulation with **(A)** TCM 12495, **(B)** TCM 24045, **(C)** TCM 17404, **(D)** TCM 43709, **(E)** TCM 18935, and **(F)** PRD_002214 (inhibitor N3 from 6LU7 co-crystal structure) using the 2D kernel density.

The MD simulation states that the loop regions are physically playing the role of protein flexibility. The structure visualization shows that the active site residues Phe140, Gly143, Cys145, Gln189, and Thr190 are present in the loop regions and those are mechanistically essential for protein function. Especially the residues between the 132 and 146 AA regions are unstructured loops and may undergo partial folding with the binding of the substrate to initiate the transition state. The conformational changes from SARS-CoV-2 M^pro^ are a core mechanism, which makes us consider the ensemble docking methods for virtual screening. For the ensemble docking approach, 21 conformations are taken as stated in the materials and methods, and those conformations are aligned, as shown in Figure 3A. Each conformation of the RMSD values is plotted in a heat map by applying the identity matrix method, as provided in Figure 3B. The heat map generated from 20 × 20-based RMSD values visualizes all the pairwise correlations showing the RMSD values in the 0.36–0.71-nm range. The color codes of heat map in red indicate the values between 0.42 and 0.71 nm; blue color indicates values of 0–0.28 nm and the remaining mid regions in white. The heat map in Figure 3B shows a deep red color range from 0.55 to 0.71 nm, and the light red color below 0.50 nm indicates that a variety of conformations are adopted, illustrating the degree of conformational diversity. Thus, with a variety of conformations, along with average conformation, the 21 conformation-based grids are prepared and allowed to dock with a prepared TCM database. The crystal structure of SARS-CoV-2 M^pro^ complexed with an N3 inhibitor yields a docking score of −7.63 kcal/mol and binding energy of −52.38 kcal/mol. Based on this, we have assigned the scrutinization filter of choosing the compounds showing a minimum docking score of −8.00 kcal/mol and binding energy of −53.00 kcal/mol with all 21 conformations extracted from MD simulations. The final XP docking with the TCM database provides the compounds TCM 12495, TCM 24045, TCM 17404, and TCM 43709 with these filtering criteria, and an another compound TCM 18935 shows potential but with one conformation, which shows the docking score −7.86 kcal/mol. Apart from these five compounds, few other hit compounds are eliminated due to lack of interactions with the core active site residues. The final average scoring values (mean value of 21 poses) of hit compounds and co-crystal ligand are provided in Supplementary Table 2. For the known compound, the average docking score is −7.99 kcal/mol, and the average binding energy is −60.172 kcal/mol, but the hit compounds have the tendency to surpass those values. Docking vs binding comparison is fitted in the linear fit model, and the *R*^2^ of the predicted model clearly represents that the compounds TCM 12495, TCM 24045, TCM 17404, TCM 43709, TCM 18935, and PRD_002214 have the values of 0.0954, 0.0014, 0.2554, 0.2231, 0.2915, and 0.0839, respectively. There is a clear correlation seen with a docking score and binding energy, especially if the binding energy is below −60 kcal/mol, when the docking score ranges between −7 and −10 kcal/mol. For representation of the docking vs binding correlation, the 2D kernel display plot is provided in Figure 4. The deep-blue regions in Figures 4A–F represent the zone of higher density (ZHD) for the correlation of docking score and binding energy. The ZHDs of hit compounds TCM 12495, TCM 24045, TCM 17404, TCM 43709, and TCM 18935 range between −8.9 and −9.3 kcal/mol, −8.7 and −9.3 kcal/mol, −8.8 and 9.4 kcal/mol, −8.7 and −9.4 kcal/mol, and −8.9 and −9.2 kcal/mol, which directly impose the binding energy density zone with the range of −72 to −76 kcal/mol, −72 to −78 kcal/mol, −61 to −65 kcal/mol, −59 to −63 kcal/mol, and −64 to −71 kcal/mol, respectively. While the N3 bound holo forms show the ZHD range of −7.9 to −8.2 kcal/mol in docking score and −59 to −63 kcal/mol in binding energy, it clearly represents the hit compounds ZHD is energetic than the available co-crystal ligand.

### Induced Fit Docking (IFD)

**Figure 5:**
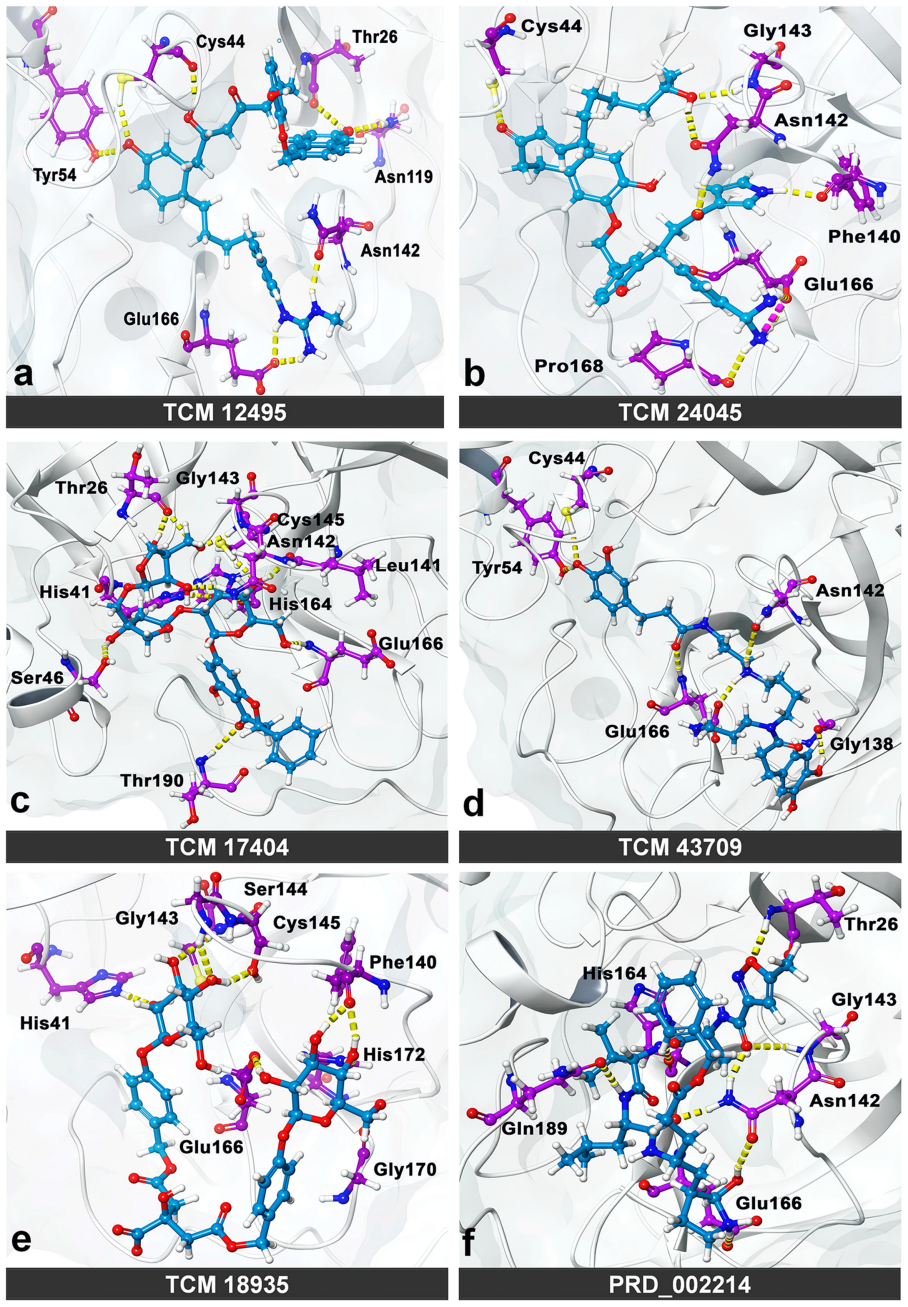
Molecular interaction of compounds screened from the TCM database: **(a)** TCM 12495, **(b)** TCM 24045, **(c)** TCM 17404, **(d)** TCM 43709, **(e)** TCM 18935, and **(f)** PRD_002214 (inhibitor N3 from 6LU7) in interaction with average conformation obtained from 1 microsecond (μs) of MD simulations through the induced fit docking (IDF) method.

While the ensemble docking approach provides better hit compounds than the co-crystal ligand, those hit compounds are redocked with the IFD method, to obtain the best pose among the multiple conformations. This IFD is purposefully performed with the average conformation (Supplementary Figure 5) of the 1-μs MD simulation, for avoiding the limitation of partial flexibility in XP docking. The IFD approach provides up to 30 possible poses per ligand and ranked based on best poses. For that, the best hit compounds (2D information available in Supplementary Figure 6) and co-crystal ligand are flexibly allowed to interact with average conformation of SARS-CoV-2 M^pro^. The IFD results seem to be interesting by showing strong bonding interactions with functionally important active sites, with strong scoring values. Like XP docking, the IFD provides the scoring values, represented in Table 2, indicating that the hit compounds surpass the available co-crystal ligand. The IFD-based docking score for the compounds TCM 12495, TCM 24045, TCM 17404, TCM 43709, TCM 18935, and PRD_002214 are −16.81, −12.40, −17.73, −14.95, −15.07, and −10.05 kcal/mol, respectively. Similarly, the IFD scores are above −660 kcal/mol for both co-crystal and hit compounds, and the best pose of interactions is shown in Figures 5a–f. The changes between XP docking and IFD are seen in scoring values along with the interactions. The co-crystal compounds show 8 hydrogen interactions with SARS-CoV-2 M^pro^ with the main support of residues Asn142 and Gly143. The hit compound, namely, TCM 17404, can form 11 hydrogen bond interactions with Asn142, Gly143, and Cys145. The other hit compound interactions also show a strong binding with the active sites; the details of interactions are provided in Supplementary Table 3. We expect the hit compounds to interact with Phe140, Gly143, Cys145, His163, His164, Glu166, Gln189, and Thr190 residues in resembling the co-crystal ligand binding. However, the hit compounds interact with core functional residues His41, Gly143, and Cys145, along with Thr26, Ser46, Tyr54, Asn119, Gly138, Phe140, Leu141, Asn142, His163, His164, Glu166, Pro168, Gly170, His172, Gln189, and Thr190. For conforming these residues' involvement in experimental structures, these residues are cross-checked with available crystal structure interactions provided in Supplementary Table 1. Interestingly, the residues interacted with hit compounds are experimentally reported to have the bonding interactions.

**Table 2:** IFD scores of new and known compounds with an average structure of M^pro^/3CL^pro^ obtained from 1 μs MD simulation.

Compound name/ID*	**Docking score (kcal/mol)**	**IFD score (kcal/mol)**	**No of H. bonds**	**Atomic interactions**	**Π-Π interaction**
TCM 12495	−16.8	−671.8	07	Supplementary Table 3	0
TCM 24045	−12.4	−669.0	08		1
TCM 20302	−17.7	−667.6	11		0
TCM 43709	−14.9	−666.4	06		2
TCM 18935	−15.0	−662.2	09		0
PRD_002214	−10.0	−667.1	08		0

### Molecular Dynamics Simulation: Stage II

**Figure 6:**
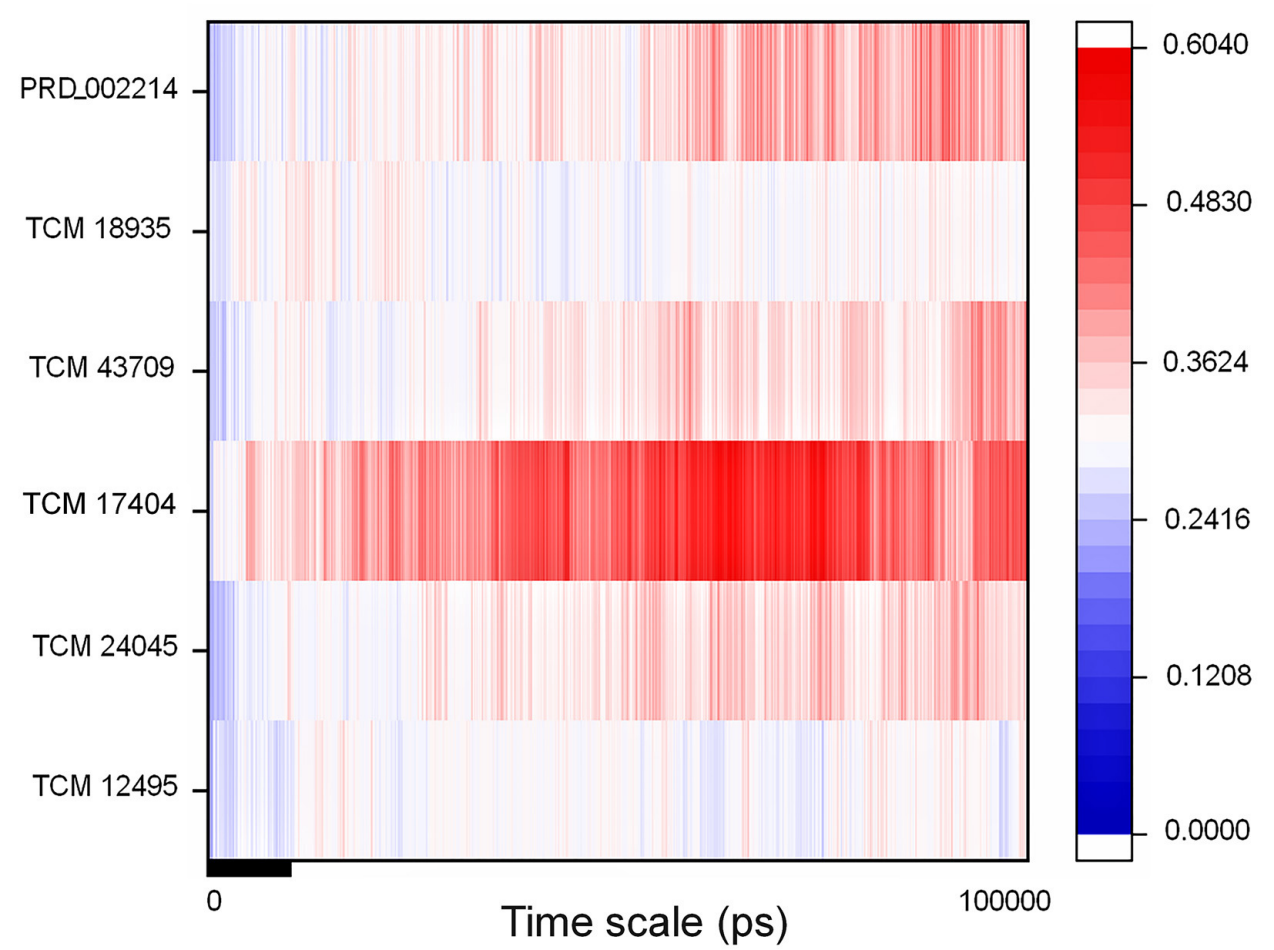
RMSD of the SARS-CoV-2 main protease (M^pro^/3CL^pro^) with respect to inhibitor binding in the time scale of 100 nanoseconds (ns) of MD simulation; here blue indicates the least value close to zero and red indicates higher value <0.65.

The IFD complex shows prominence in binding and scoring parameters, and those final poses are simulated for 100 ns (stage II MD) for understanding the ligand stability in dynamic state. Here the IFD best pose for hit compound and co-crystal ligand stability is concerned, as the whole workflow relies on multiple protein conformations. This ligand stability in the 100-ns MD simulations can conform to the hit compound binding efficiency in the dynamic state. The RMSD plot for the ligand-bound complex is provided in Supplementary Figure 7, which shows that only the TCM 17404 complex shows the RMSD above 0.4 nm. Except the TCM 17404 complex, all the other hit compounds and co-crystal bound ligands show the RMSD values below 0.4 nm. Each snapshot deviation is calculated and plotted in Figure 6; the color codes of blue represent the values below 0.25 nm, those in red color represent the values above 0.35 nm, and the range between 0.25 and 0.34 nm is represented in white. The hit compound TCM 17404, showing the deviations from the 5^th^ to 100^th^ ns with dominant red color, indicates higher RMSD values. The hit compounds TCM 24045 and TCM 43709 and the co-crystal ligand (PRD_002214) complex show a moderate red color, as the RMSD lies between 0.25 and 0.4 nm. The other hit compound, TCM 18935 complex, has shown limited deviations, with blue and white color lines indicating the RMSD ranges between 0.24 and 0.36 nm.

**Figure 7:**
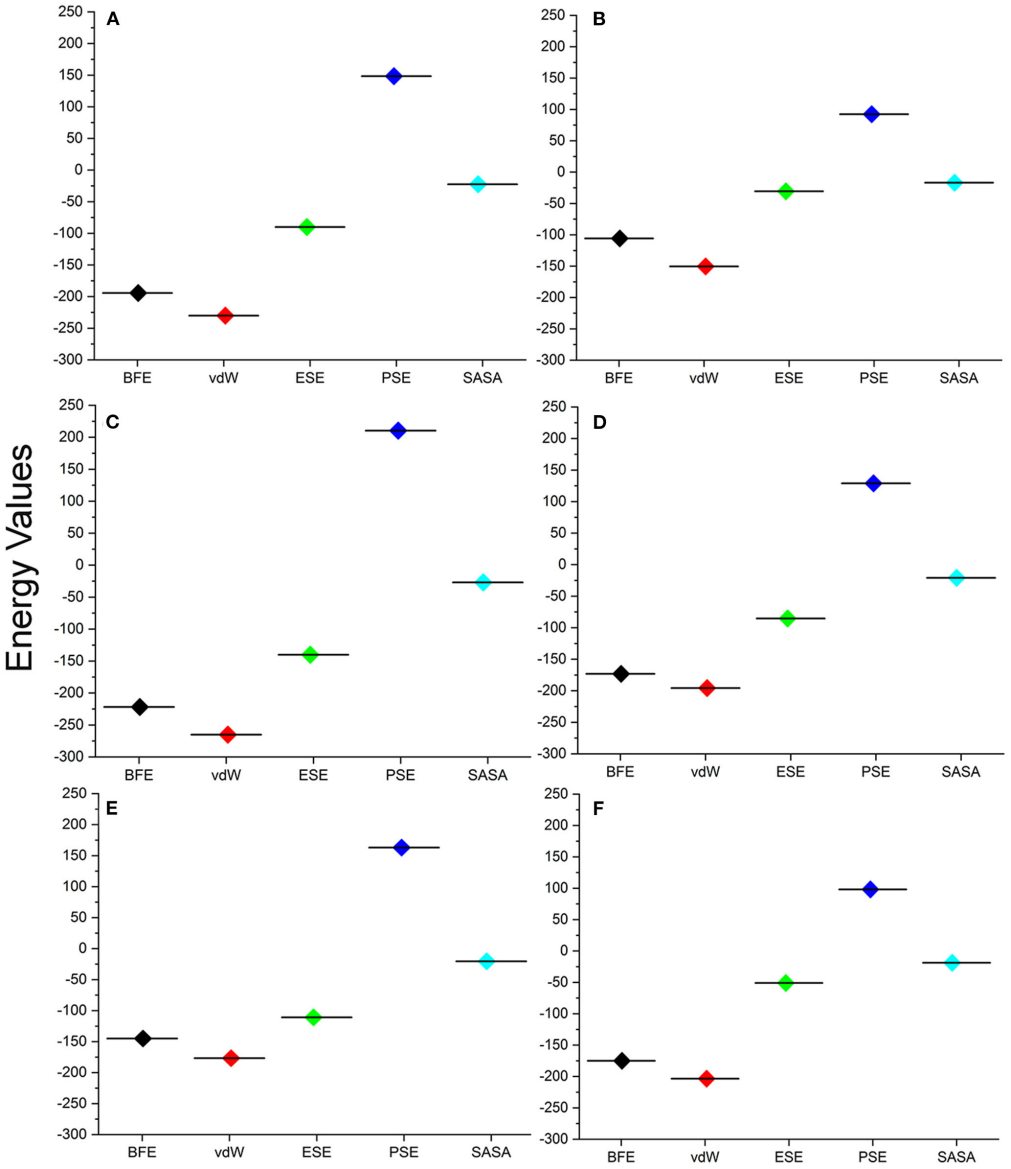
Energy values obtained from the MM/PBSA script for the screened compounds [**(A)** TCM 12495, **(B)** TCM 24045, **(C)** TCM 17404, **(D)** TCM 43709, **(E)** TCM 18935] and from inhibitor N3 **(F)** from the SARS-CoV-2 co-crystal structure, PDB ID:6LU7 for the simulation timescale of 100 ns.

For understanding these RMSD values, the statistical methods are applied and incorporated in Table 3. Even though the range (min to max) of compounds, namely, TCM 12495 (a), TCM 24045 (b), TCM 17404 (c), TCM 43709 (d), TCM 18935 (e), and PRD_002214 (f) are 0.1–0.38, 0.−0.43, 0.1–0.60, 0.1–0.47, 0.1–0.38, and 0.1–0.49, respectively, about 95% of the data of compounds a–f, lies in the interval 0.25–0.33, 0.26–0.39, 0.30–0.57, 0.25–0.39, 0.26–0.34, and 0.25–0.43, respectively. The mean, median, and mode are approximately equal for all compounds except for compound c. This shows that, except compound c, all the value distributions of the compounds can be assumed to be approximately symmetrical. The compound c mode is lesser than the mean and median, so that the value distribution of compound c is skewed to the high deviation. On analyzing the standard deviation of the RMSD values of the protein–ligand complex, all the compounds show the values approximately equal to zero. Through this, we understood that 100-ns MD simulations produced RMSD values approximately identical, which is equal to its mean value. The coefficients of variations for compounds a, b, c, d, e, and f are 0.06, 0.10, 0.15, 0.10, 0.06, and 0.13 nm, respectively. In this, compounds a (TCM 12495) and e (TCM 18935) are holding smaller coefficients of variations than the others. So, compounds a and e can be more consistent than the others. For validation of protein–ligand interaction, various energy values are analyzed which contributed to the efficient binding. Through MM/PBSA calculations, binding energy (BFE), van der Waals (vdW), electrostatic energy (ESE), polar solvent energy (PSE), and SASA energy are predicted and the values are provided in Table 4 and Figures 7A–F. From the screened compounds, TCM 12495, TCM 17404, and TCM 43709 show lower binding energy values of −194.262 kJ/mol, −221.947 kJ/mol, and −173.163 kJ/mol, respectively. This shows that the compounds TCM 12495, TCM 17404, and TCM 43709 have a higher stability in the 100-ns MD simulations. Other compounds, namely, TCM 24045 and TCM 18935, also show moderate binding energy that resembles known co-crystal ligands. For attaining these energy levels, the other energy parameters like van der Waals (vdW), electrostatic energy (ESE), and SASA energy have contributed to supporting the ligand stability. In comparing all the energies, the polar solvation energy (PSE) is the only energy which contributed positively to the total binding free energy. Thus, the predicted binding energy strongly supports the binding interactions of an effective compound with the targeted protein. The validation of the virtual screening-based docking protocol was evaluated using the enrichment calculation method with EF, receiver operating characteristic (ROC), and Boltzmann-Enhanced Discrimination of the receiver operating characteristic (BEDROC) metrics. ROC scores is received with 0.98 RIE of 15.18, respectively, which represents the higher-ranking order of active compounds based on quality. Because ROC score with “≥ 0.7” signifies a satisfied metric value to define the highest precision and predicting skill of virtual screening and docking protocol defined. Supplementary Table 4 shows the enrichment metric value, and the graphical representation enrichment curve in Supplementary Figure 8 represents the quality of retrieval of known actives from the external database, which were ranked to decoys for evaluation.

**Table 3:** Statistical analysis of RMSD values obtained from the 100 ns of MD simulation for screened compounds for comparing the stability with co-crystal compound PRD_002214.

**Compound**	**Mean**	**Median**	**Mode**	**SD**	**Variance**	**Coefficient of variation**	**Min**	**Max**
TCM 12495	0.29	0.30	0.28	0.02	0.00	0.06	0.1	0.38
TCM 24045	0.32	0.33	0.33	0.03	0.00	0.10	0.1	0.43
TCM 20302	0.43	0.44	0.32	0.06	0.00	0.15	0.1	0.60
TCM 43709	0.32	0.32	0.28	0.03	0.00	0.10	0.1	0.47
TCM 18935	0.30	0.30	0.28	0.01	0.00	0.06	0.1	0.38
PRD_002214	0.34	0.33	0.27	0.04	0.00	0.13	0.1	0.49

**Table 4:** Energy values obtained from the MM/PBSA script for the screened compounds and co-crystal compound for the simulation timescale of 100 ns.

**S. No**.	**Compound ID**	**Binding energy (kJ/mol)**	**van der Waal energy (kJ/mol)**	**Electrostatic energy (kJ/mol)**	**Polar solvation energy (kJ/mol)**	**SASA energy (kJ/mol)**
1	TCM12495	−194.2 ± 33.0	−230.0 ± 27.7	−90.0 ± 33.8	148.4 ± 20.4	−22.5 ± 2.4
2	TCM24045	−105.7 ± 25.6	−150.4 ± 22.8	−30.6 ± 22.5	92.3 ± 24.3	−16.9 ± 2.9
3	TCM20302	−221.9 ± 35.5	−265.1 ± 26.5	−140.2 ± 67.4	210.3 ± 45.5	−26.9 ± 2.1
4	TCM43709	−173.1 ± 51.3	−195.7 ± 50.2	−85.3 ± 48.6	128.9 ± 31.9	−20.9 ± 4.7
5	TCM18935	−144.9 ± 47.5	−176.6 ± 24.6	−110.9 ± 63.6	163.0 ± 41.0	−20.4 ± 2.6
6	PRD_002214	−175.1 ± 20.2	−203.5 ± 17.6	−50.9 ± 19.8	98.1 ± 15.1	−18.7 ± 1.6

*Units (kJ/mol)*.

## Discussion

The enzyme SARS-CoV-2 M^pro^ is known to control the activity of the replication mechanism and is designated as an attractive target for inhibition. We have applied the MD simulations for elucidating the functional active site role in M^pro^ along with conformational changes, ligand-binding effect, and stability with the apo and holo forms. Long-scale MD simulation shows that the apo form of SARS-CoV-2 M^pro^ shows higher fluctuations than the holo form. The whole conformational heterogeneity shown by the binding site loop in the apo form persists to a large degree of deviations in the 1-μs MD simulations. The RMSF and statistical analysis shows that the functional residues in apo form are key to representing the fluctuations in the MD simulations. For the holo forms, those functional residues grasp the ligand molecule, steady its positions, and show stability throughout the MD simulations. Yoshino *et al*. have also reported the high-end MD simulations, to show the importance of the roles of His41, Gly143, and Gly166 in peptide functional groups (Yoshino et al., 2020). Similarly, the co-crystal ligand (PRD_002214) shows the binding sites of Phe140, Gly143, Cys145, His163, His164, Glu166, Gln189, and Thr190, and from this, except for Phe140 and Gly143, all the other amino acids are polar and charged. These charged residues play a vital role in electron transfer and modulate the overall electrostatic balance of the protein and its binding. The functional behavior of apo protein which leads to multiple conformations is controlled by binding of a suitable inhibitor between the two anti-parallel β-barrel domains that result in stable protein–ligand MD simulations. This may be due to structural changes occurring in the active site loop, also considering that we applied ensemble docking methods to employ the identification of new molecules.

Thereby the MD trajectories are processed to obtain an ensemble of distant conformations rather than a minimally fluctuating single global-minima structure. Perhaps an active site is located in between the two anti-parallel β-barrel domains, especially the loop regions as a target region of action for inhibitory molecules, which contains the catalytic Cys-His dyad. Here the electrostatic, stabilized Cys145 is a potent nucleophile linked with the nearby histidine amino acid along with the amide backbone of Cys145 and Gly143 which stabilize the oxyanion hole in the transition state formation. The His41 imidazole ring places the active site linked with Cys145 through the stabilization of thiolate ion. Nucleophilic attack of the anionic cysteine S (thiolate ion) occurs on the peptide carbonyl carbon. In this step, a fragment of the substrate is released with an (amino) amine group in the terminus, the histidine residue in the protease is restored to its deprotonated form, and a thioester intermediate linking the new carboxy-terminus of the substrate to the cysteine thiol is formed. Thus, finding an appropriate compound, which binds in the binding site residues of His41, Gly143, and Cys145, will block the substrate-binding activity. Considering the catalytic activity of His41, Gly143, and Cys145, the multiple conformation-based ensembles docking with filtering criteria of docking score of −8.00 kcal/mol and binding energy of −53.00 kcal/mol readily which threw most of the compounds from the TCM database are executed. From the whole TCM database, our screening scrutinization filters actively pass only five compounds, namely, TCM 12495, TCM 24045, TCM 17404, TCM 43709, and TCM 18935. Ensemble conformations obtained from different intervals show variations in pose and space in the binding site and may also have a functional difference. The successive compounds TCM 12495, TCM 24045, TCM 17404, TCM 43709, and TCM 18935 hold the tendency to bind multiple conformations which adapt well inside the protease active site located in between the two anti-parallel β-barrel domains, like co-crystal ligand (PRD_002214) binding. Thus, we believe that these compounds can affect the M^pro^ functional mechanism, by interacting with core active site residues.

The residual interactions of new leads in the flexible environment are shown using the re-docking method of IFD, which provides the strong support for new compounds, which are bound more efficiently than the co-crystal ligand. Normal XP docking with multiple protein conformations followed by allowing adjustments to the receptor conformation through the soft receptor approach using flexible side chains or IFD shows huge improvement. Through IFD, we have seen new compounds from the screening able to bind with functional catalytic activity residues His41, Gly143, and Cys145. Ensemble docking with multiple conformation proteins followed by IFD-derived ensembles provides high benefit of success in virtual screening. Through this, the shortlisted TCM compounds from this study and co-crystal ligand bind to the substrate-binding site in similar binding mode and will efficiently inhibit SARS-CoV-2 M^pro^ activity as shown in Supplementary Figure 9 (Dai et al., 2020; Jin et al., 2020; Zhang et al., 2020).

The screened compounds dynamic behavior is analyzed for low energy profiles using MM/PBSA, which is used for post-processing of docked structures along with the reliability of compound binding inside the flexible binding pocket. The 100 ns of protein-ligand complex simulation along with MM-PBSA binding free energy suggests that lead molecules perfectly fit in the binding site and are structurally stable with a low energy profile. Available literature suggests that the compounds with lower energy profiles in MM/PBSA are suitable candidates for further experimental analysis. In addition, the external validation with random decoy set along with the screened compounds favors the top screened compounds by positioning those compounds in the top with the ROC curve score of 0.98, which clearly shows that the screened compounds are best among the random decoy set. The successive compounds from this work are readily available as the TCM herbal compounds are believed to be non-toxic or less toxic and have been used to treat numerous kinds of diseases for more than 2,000 years in eastern Asian countries (Yuan et al., 2016). By interest, we searched the source of the screened compounds and found that the compound TCM 12495 is from the herb *Lantana camara*, and compound TCM 17404 is isolated from the herb *Viscum angulatum* (Xu et al., 2019). However, the source of TCM 18935, TCM 24045, and TCM 43709 are not available in the literature.

## Conclusion

Overall, this study provides a comprehensive structural analysis of the SARS-CoV-2 main protease (M^pro^/3CL^pro^) substrate-binding pocket for the purpose of inhibitor screening anddesign. Through this work, we have disclosed a long-range 1-μs MD simulation for the apo and holo forms of SARS-CoV-2 M^pro^. The MD simulations revealed conformational changes in the active site loop regions and, accounting those changes into the screening, provide a strong, potential compound against SARS-CoV-2 M^pro^. Small molecules targeting this binding pocket should have the ability to interact with residues His41, Gly143, and Cys145; disturbing the formation of the oxyanion hole can lead to its inhibition. The final antiviral inhibitors screened from the world's largest traditional Chinese medicine database (TCM@Taiwan) have robust scoring values that have been evaluated from the theoretical, statistical, and internal motion of atoms in dynamic status. These prospective compounds are from natural resources and used as medicine for several years, and this incorporating international effort can bring these compounds to light as suitable drug candidates against the COVID-19.

## Data Availability Statement

The datasets presented in this study can be found in online repositories. The names of the repository/repositories and accession number(s) can be found in the article/Supplementary Material.

## Author Contributions

CS, VKD, and SKS conceived and designed the study. CS and UP performed the screening and MD simulations. CS, DCD, EB, PS, VKD, and SKS analyzed the results. CS and DCD prepared the manuscript. All authors read and approved the final manuscript.

## Conflict of Interest

The authors declare that the research was conducted in the absence of any commercial or financial relationships that could be construed as a potential conflict of interest.
