# Supplementary material for: Microsecond MD Simulation and Multiple-Conformation Virtual Screening to Identify Potential Anti-COVID-19 Inhibitors Against SARS-CoV-2 Main Protease
Source: Front Chem. 2021 Jan 13;8:595273. doi: 10.3389/fchem.2020.595273 (PMC7873971; doi:10.3389/fchem.2020.595273)
Supplement: Supplementary file 1 [file Data_Sheet_1.docx]

Microsecond MD Simulation and Multiple-Conformation Virtual Screening to Identify Potential Anti-COVID-19 Inhibitors Against SARS-CoV-2 Main Protease

**Supplementary Information**

Detailed Methodology of MM/PBSA and PCA analysis

MM-PBSA – free binding energy calculation

The binding free energy of each protein-ligand complex was predicted using the g_mmpbsa tool (Molecular Mechanics Poisson-Boltzmann Surface Area) in GROMACS. Using g_mmpbsa, the molecular mechanics potential energy along with the free energy of solvation of individual complexes was analyzed as per the following equations.

ΔG_binding_ = G_complex_ – (G_protein_ + G_ligand_)

Where, G-complex represents the total free energy of the protein–ligand complexes and G-protein + G-ligand as total free energies of the separated form of protein and ligand in solvent, respectively.

ΔG_binding_ = E_gas_ + G_sol_ − TΔS

E_gas_ = E_int_ + E_vdw_ + E_ele_

G_sol_ = G_pol_ + G_non-pol_

G_nonpol_ = γSASA + β

Herein, the overall energy ΔG_binding_ for each (complex, ligand, or receptor) can be decomposed by a gas phase energy (E_gas_), solvation energy (G_sol_) and an entropy term (TΔS). In which, E_gas_ is a sum of the internal energy of bonds (E_int_) and non-bonds (E_vdw_ & E_ele_), G_sol is_ part of polar (G_pol_) and nonpolar (G_non-pol_) energies. Both G_pol_ and G_non-pol_ were determined by generalized Born (GB) equation and solvent accessible surface area, respectively. An entropy term (TΔS) includes various terms of translational, rotational, and vibrational of the solute molecules.

**Table S1: Molecular interactions of compounds in the crystal structure of Main protease**

| PDB ID | Ligand | Molecular Interactions | Residues Involved |
| --- | --- | --- | --- |
| 6XMK | 7j | 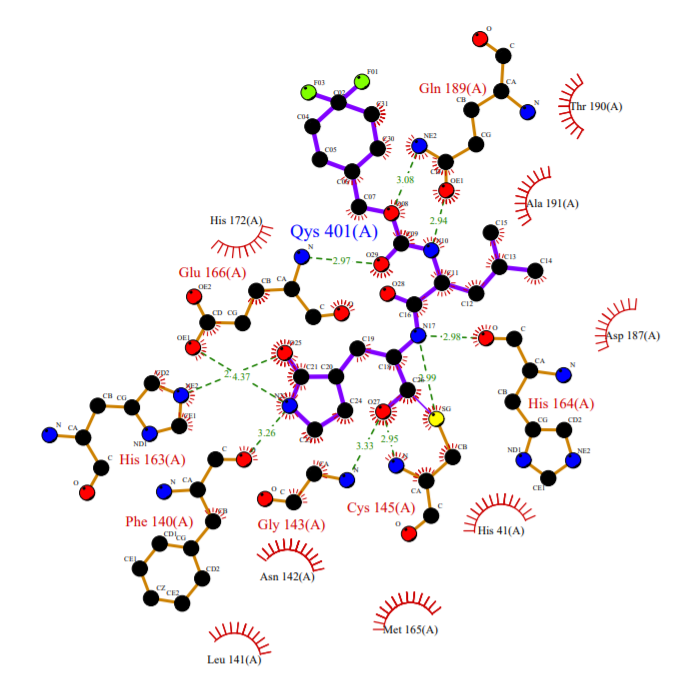 | Gln189, Glu166, His164, His163, Phe140, Gly143, Cys145 |
| 6Y2G | O6K | 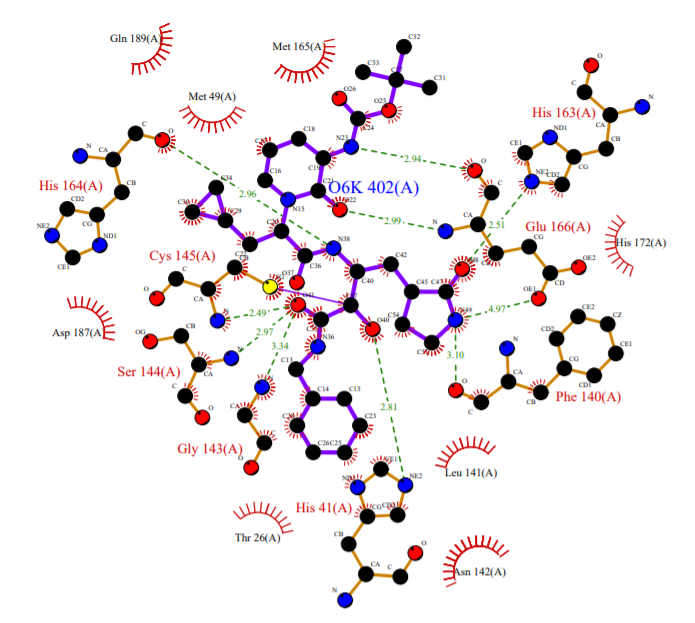 | His163, His164, Glu166, Cys145, Ser144, Gly143, His41, Phe140, Glu166 |
| 6Y2F | O6K | 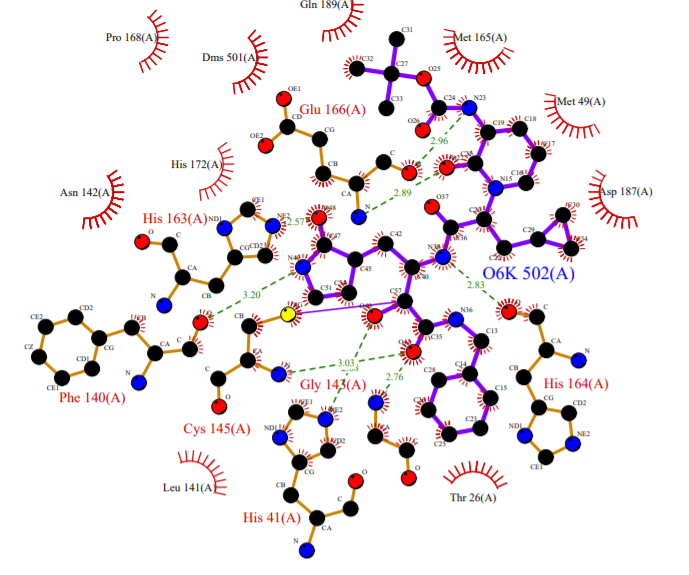 | Glu166, His164, His41, Gly143, Cys145, Phe140, His163 |
|  |  |  |  |
| 6XFN | UAW243 | 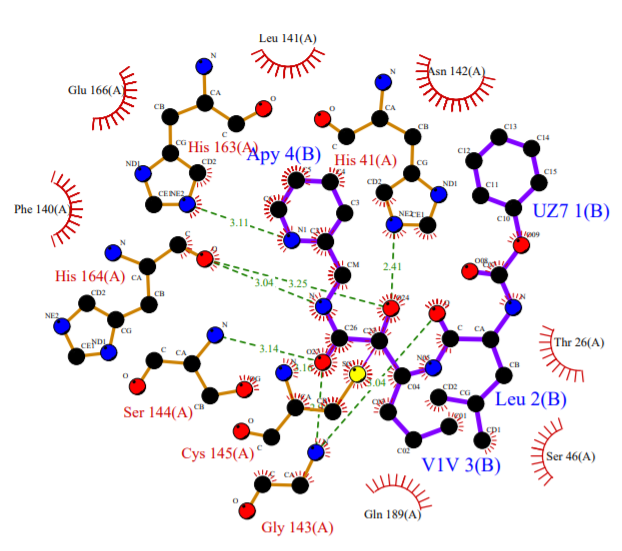 | His163, His41, Gly143, Cys145, Ser144, His164 |
| 6ZRT | Telaprevir | 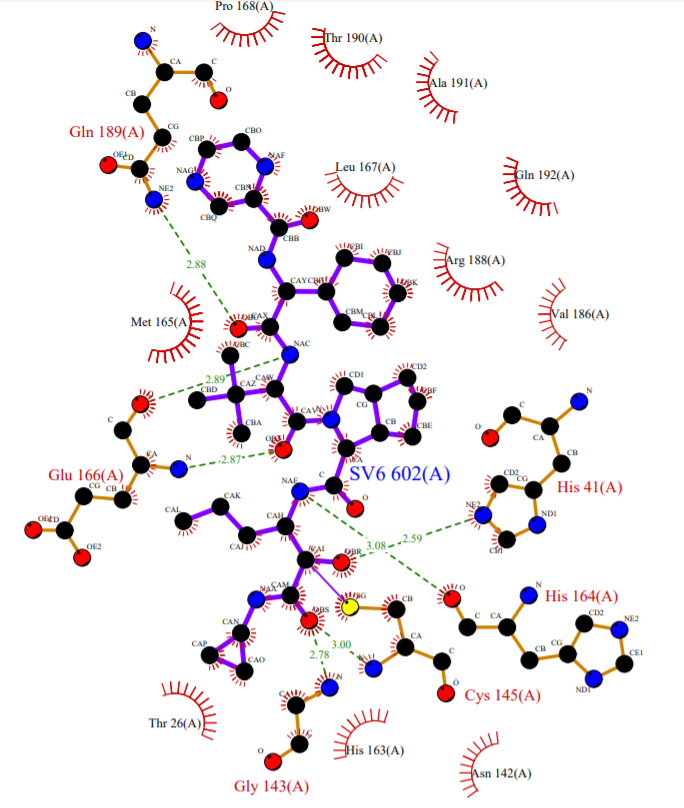 | Gln189, Glu166, Gly143, Cys145, His164, His41 |
| 6ZRU | Boceprevir | 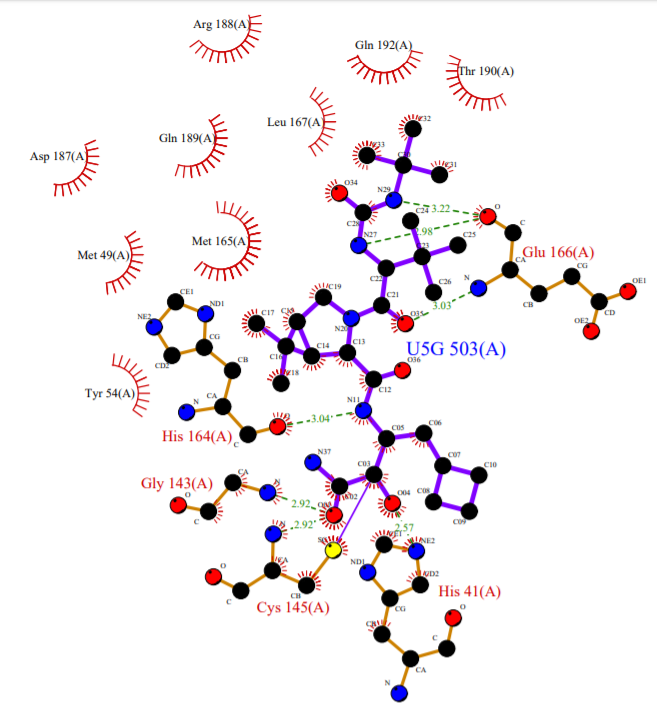 | Glu166, His164, Gly143, Cys145, His41 |
|  |  |  |  |
| 7BQY | N3 | 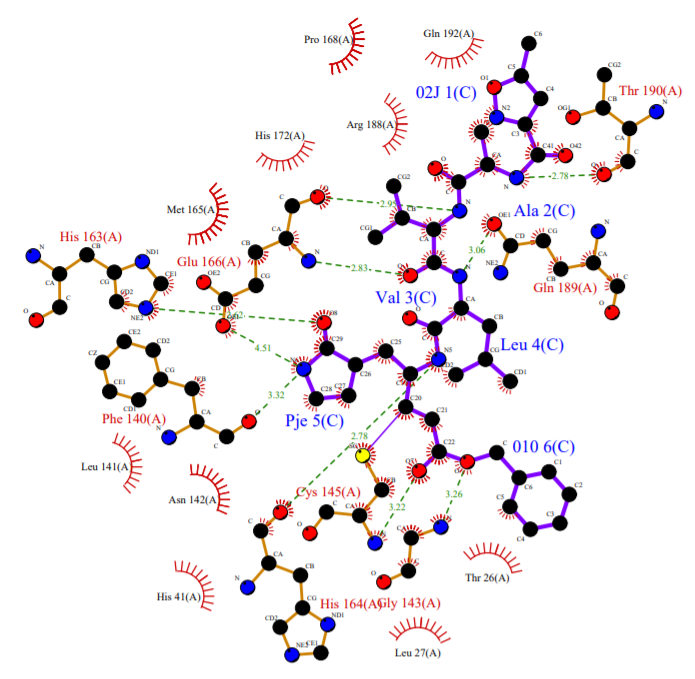 | Thr190, Gln189, Gly143, Cys145, His164, Phe140, His163 |
| 7BRP | Boceprevir | 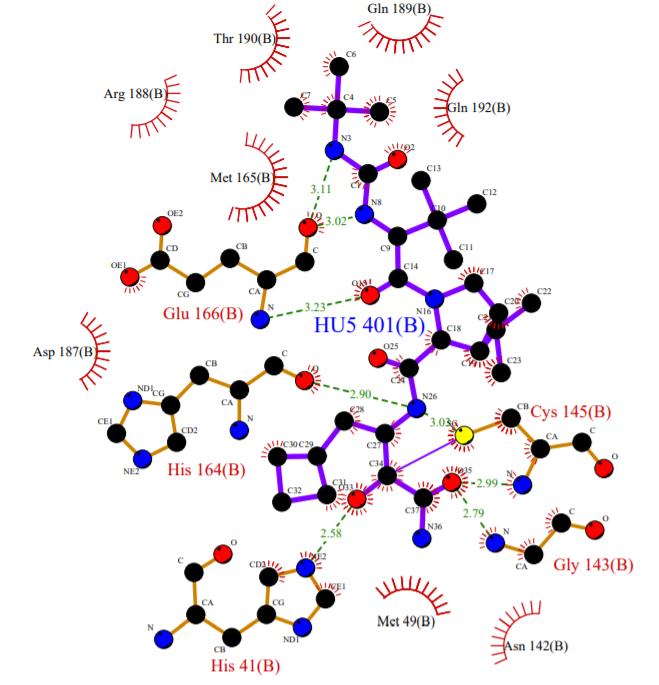 | Glu166, His164, His41, Cys145, Gly143 |
| 7BUY | carmofur | 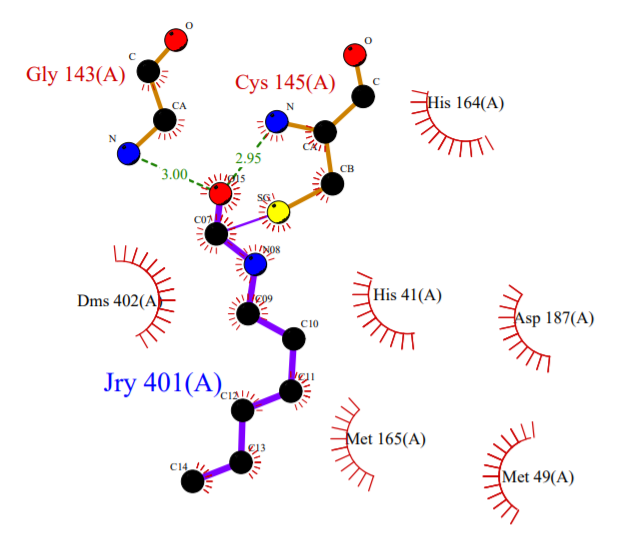 | Gly143, Cys145 |
|  |  |  |  |
| 6LZE | 11a | 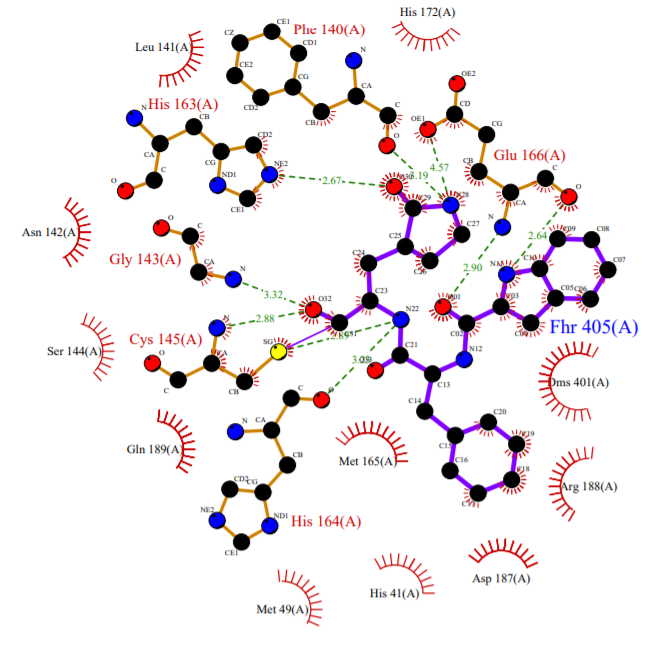 | Phe140, Glu166, His163, Gly143, Cys145, His164 |
| 6M0K | 11b | 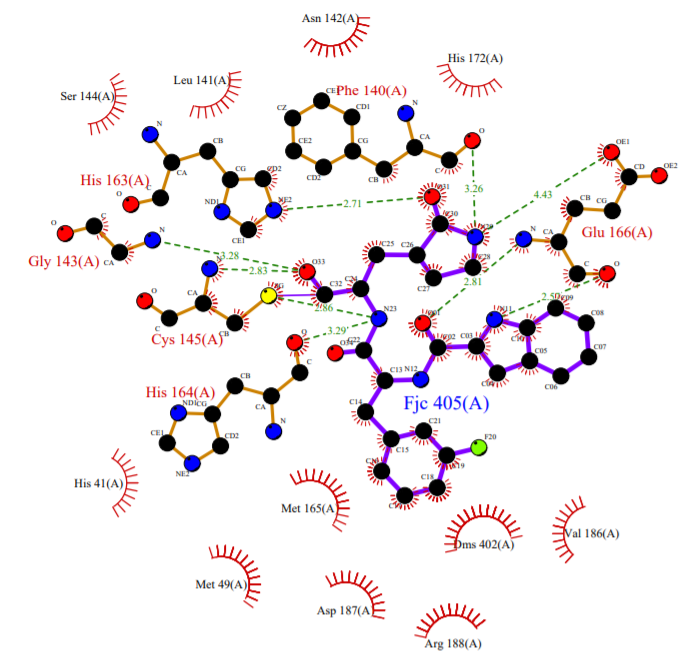 | Phe140, His163, Gly143, Cys145, His164, Gly166 |

TABLE S2. Linear fit statistical model for the relationship values between docking vs binding energy

| Compound Name | Average Docking Score | Average Binding Energy | R-Square | Y= a + bX |
| --- | --- | --- | --- | --- |
| TCM 12495 | -8.9385 | -72.097 | 0.0954 | 2.6653x - 48.273 |
| TCM 24045 | -9.0465 | -73.2585 | 0.0014 | 0.5563x - 68.226 |
| TCM 20302 | -9.1785 | -62.7315 | 0.2554 | 3.2683x - 32.734 |
| TCM 43709 | -9.232 | -61.707 | 0.2231 | 1.9906x - 43.33 |
| TCM 18935 | -8.758 | -65.598 | 0.2915 | 7.766x + 2.4167 |
| PRD_002214 | -7.9935 | -60.172 | 0.0839 | 3.7824x - 29.937 |

Table S3: Atomic interactions of protein and ligand in connection with Table 2

| Compound Name | No. of H-bond | Protein-Ligand H-bond Interaction | Distance in Angstrom | | Π-Π Interaction |
| --- | --- | --- | --- | --- | --- |
| TCM 12495 | 07 | Thr26 O(366)A ….. Lig H(4766)D | 2.31 | | No |
|  |  | Cys44 O(660)A ….. Lig H(4752)D | 1.99 | |  |
|  |  | Tyr54 H(667)D ….. Lig O(4727)A | 2.25 | |  |
|  |  | Asn119 H(1874)D ….. Lig O(4715)A | 2.26 | |  |
|  |  | Asn142 OD1(2208)A ….. Lig H(4778)D | 1.98 | |  |
|  |  | Glu166 OE1(2208)A ….. Lig H(4777)D | 1.98 | |  |
|  |  | Glu166 OE1(2208)A ….. Lig H(4783)D | 1.85 | |  |
|  | | | | | |
| TCM 24045 | 08 | Cys44 HG(667)D ….. Lig O(4697)A | 2.27 | | Yes |
|  |  | Phe140 O(2166)A ….. Lig H(4781)D | 1.86 | |  |
|  |  | Asn142 HD22(2214)D ….. Lig O(4723)A | 1.92 | |  |
|  |  | Asn142 OD1 (2208)A ….. Lig H(4774)D | 2.02 | |  |
|  |  | Gly143 H(2220)D ….. Lig O(4731)A | 2.11 | |  |
|  |  | Glu166 OE2(2550)A ….. Lig H(4761)D | 1.95 | |  |
|  |  | Glu166 OE2(2550)A ….. Lig H(4762)D | 1.87 | |  |
|  |  | Pro168 O(2579)A ….. Lig H(4760)D | 2.23 | |  |
|  | | | | | |
| TCM 20302 | 11 | Thr26 O(366)A….. Lig H(4765)D | 2.38 | | No |
|  |  | Thr26 O(366)A….. Lig H(4764)D | 1.82 | |  |
|  |  | His41 HE2(621)D….. Lig H(4723)A | 1.77 | |  |
|  |  | Ser46 OG(687)A…..Lig H(4767)D | 2.05 | |  |
|  |  | Leu141 O(2186)A…..Lig H(4759)D | 1.91 | |  |
|  |  | Asn142 HD21(2215)D…..Lig O(4728)A | 2.00 | |  |
|  |  | Gly143 H (2220)D…..Lig O(4726)A | 1.79 | |  |
|  |  | Cys145 HG(2244)D…..Lig O(4722)A | 2.10 | |  |
|  |  | His164 O(2511)A…..Lig H(4760)D | 2.36 | |  |
|  |  | Glu166 H (2551)D…..Lig O(4725)A | 2.06 | |  |
|  |  | Thr190 H(2900)D…..Lig O(4716)A | 2.61 | |  |
|  | | | | | |
| TCM 43709 | 06 | Cys44 HG(667)D…..Lig O(4690)A | 2.23 | | Yes  (Two Pi-Pi) |
|  |  | Tyr54 OH(809)A…..Lig H(4725)D | 1.72 | |  |
|  |  | Gly138 O(2148)A…..Lig H(4753)D | 2.09 | |  |
|  |  | Asn142 OD1(2208)A…..Lig H(4763)D | 2.60 | |  |
|  |  | Glu166 H (2551)D…..Lig O(4694)A | 1.99 | |  |
|  |  | Glu166 OE2(2550)A…..Lig H(4737)D | 1.87 | |  |
|  | | | | | |
| TCM 18935 | 09 | His41 HE2(621)D…..Lig O(4732)A | 1.85 | | No |
|  |  | Phe140 O(2166)A…..Lig H(4746)D | 1.72 | |  |
|  |  | Phe140 O(2166)A…..Lig H(4749)D | 1.80 | |  |
|  |  | Gly143 H(2220)D…..Lig O(4731)A | 2.19 | |  |
|  |  | Ser144 OG(2228)A…..Lig H(4770)D | 1.97 | |  |
|  |  | Cys145 H (2240)D…..Lig O(4729)A | 2.41 | |  |
|  |  | Glu166 OE2(2550)A…..Lig H(4750)D | 1.85 | |  |
|  |  | Gly170 O(2607)A…..Lig H(4751)D | 1.82 | |  |
|  |  | His172 HE2(2643)D…..Lig O(4701)A | 2.06 | |  |
|  | | | | | |
| PRD_002214  N3 inhibitor from co-crystal structure, PDB ID: 6LU7 | 08 | Thr26 H(370)D ….. Lig O(4728)A | | 1.92 | No |
|  |  | Asn142 OD1(2208)A ….. Lig h(4783)D | | 2.20 |  |
|  |  | Asn142 HD22(2214)D ….. Lig O(4727)A | | 1.75 |  |
|  |  | Asn142 HD21(2215)D ….. Lig O(4729)A | | 1.91 |  |
|  |  | Gly143 H(2220)D…..Lig O(4729)A | | 2.24 |  |
|  |  | His164 O(2511)A…..Lig H(4781)D | | 2.52 |  |
|  |  | Glu166 OE2(2550)A…..Lig H(4784)D | | 1.65 |  |
|  |  | Gln189 OE1(2883)A…..Lig H(4780)D | | 2.06 |  |

**Table S4:** Evaluation of virtual screening-based docking protocol by enrichment calculation.

| **RIE** | **ROC** | **Area under accumulation curve** | **Enrichment**  **matric** | **Value** |
| --- | --- | --- | --- | --- |
| 15.18 | 0.98 | 0.98 | BEDROC (α=160.90)  BEDROC (α=20.00)  BEDROC (α=8.00) | 0.60  0.80  0.90 |

**Supplementary Figures**

**Figure S1**


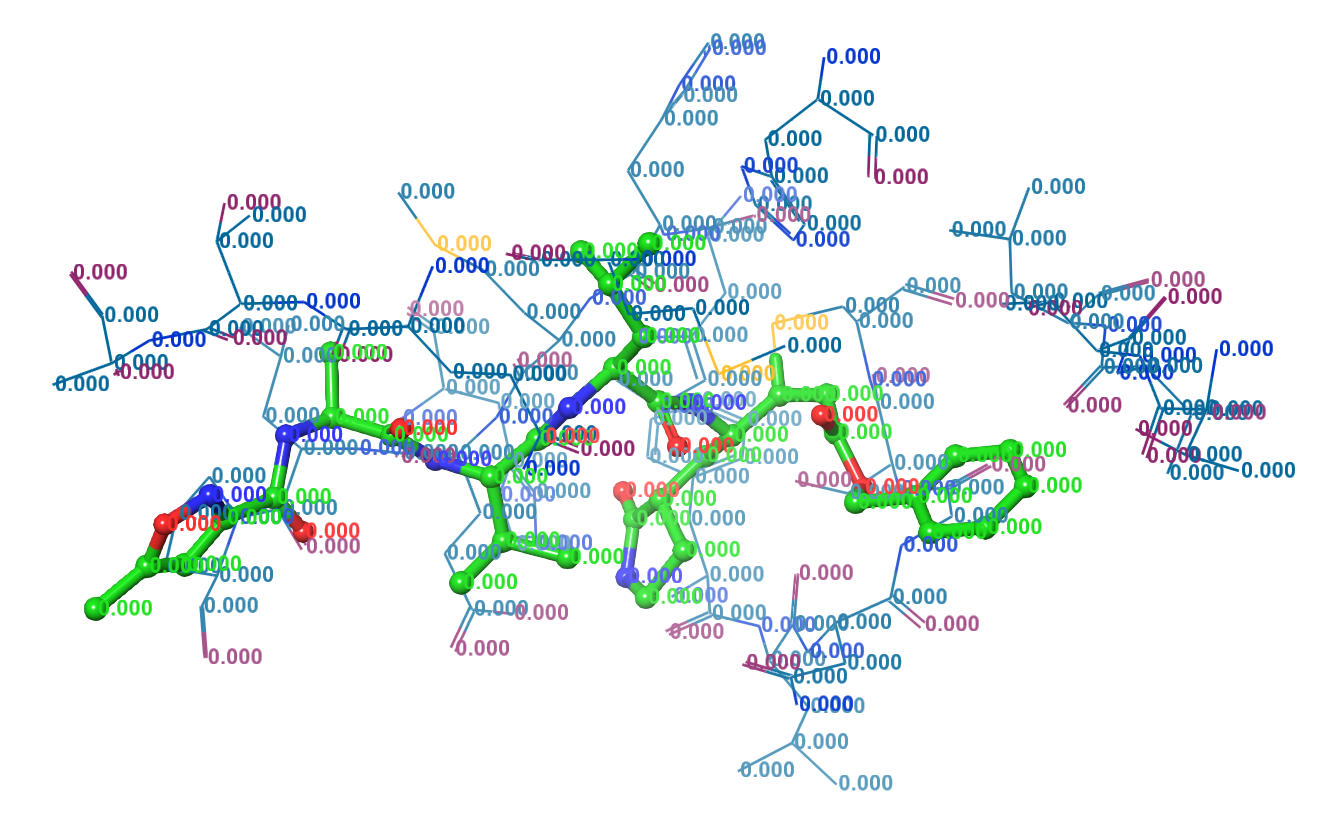

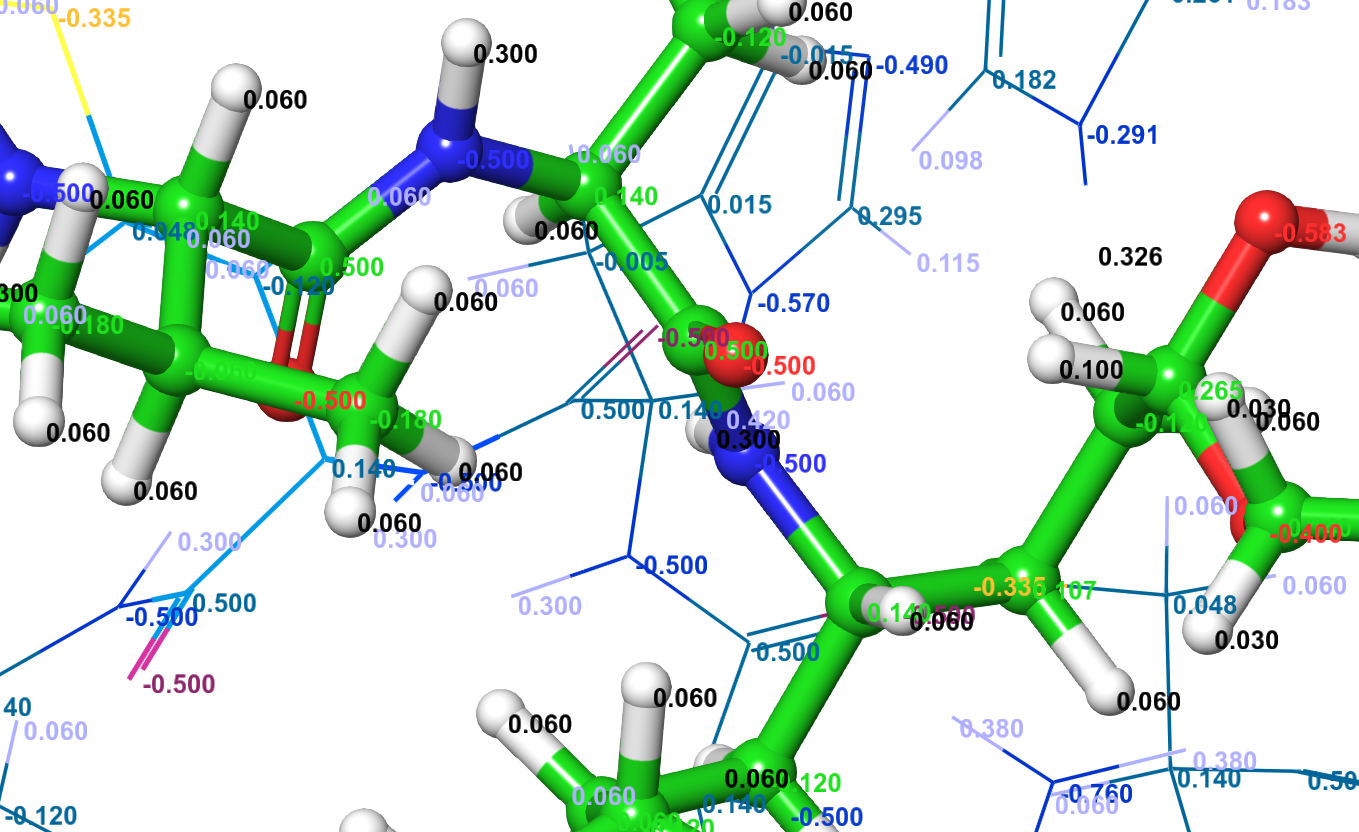


Figure S1 (a) Partial charges showing neutral charges before the protein preparation. (b) Partial charges showing the positive and negative charges after the protein preparation using the protein preparation wizard.


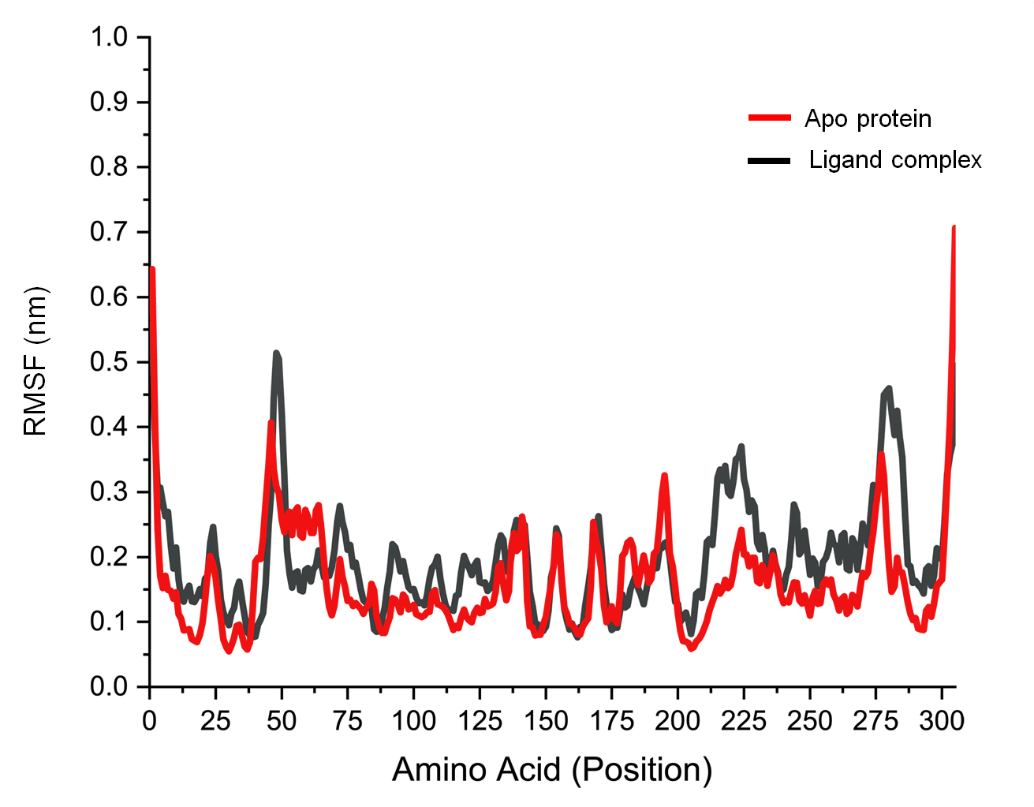


Figure S2. RMSF graph for the MD simulation for the timescale of 1 microsecond, here the apo (red) and holo (black) formsFigure S3


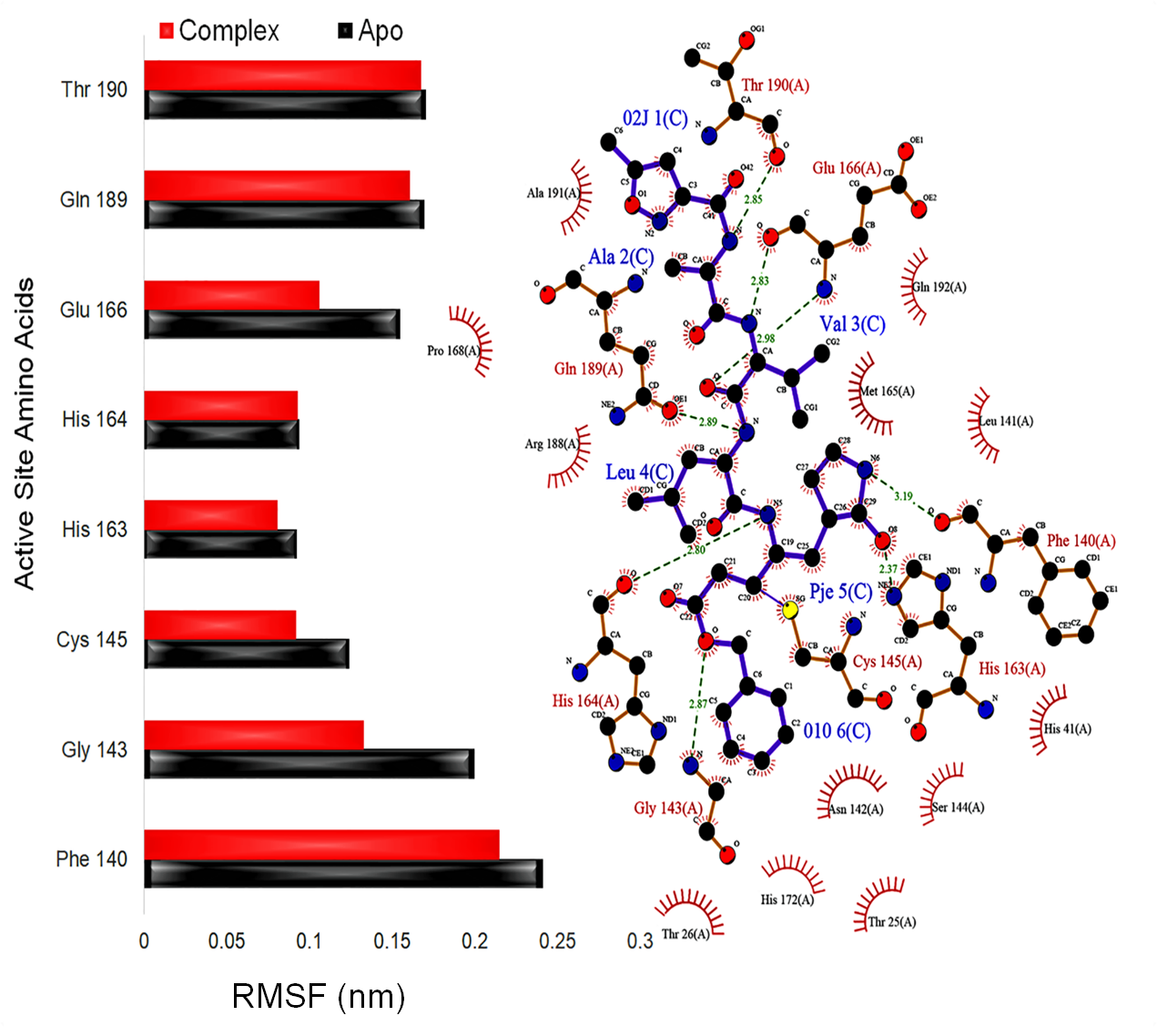


Figure S3. RMSF graph of active site amino acids interacting with inhibitor N3, here the red indicates the apo protein amino acids and black indicates the protein-ligand complex amino acids. Specific amino acids along with interacting ligand is represented in 2D Ligplot.


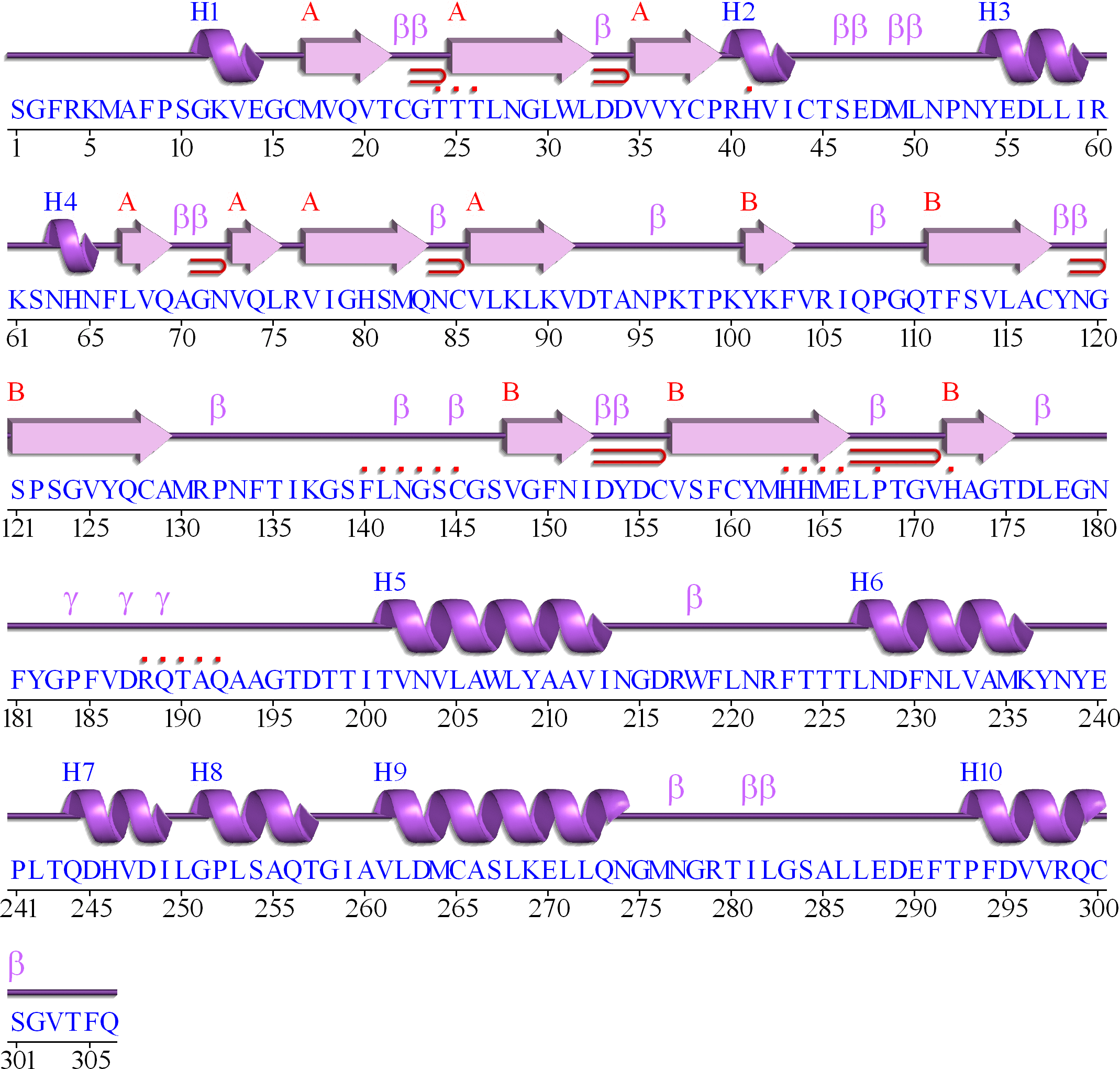


Figure S4. Secondary structure of the SARS-CoV-2 main protease (M^pro^/3CL^pro^), obtained through the PDB ID: 6LU7 from PDBsum


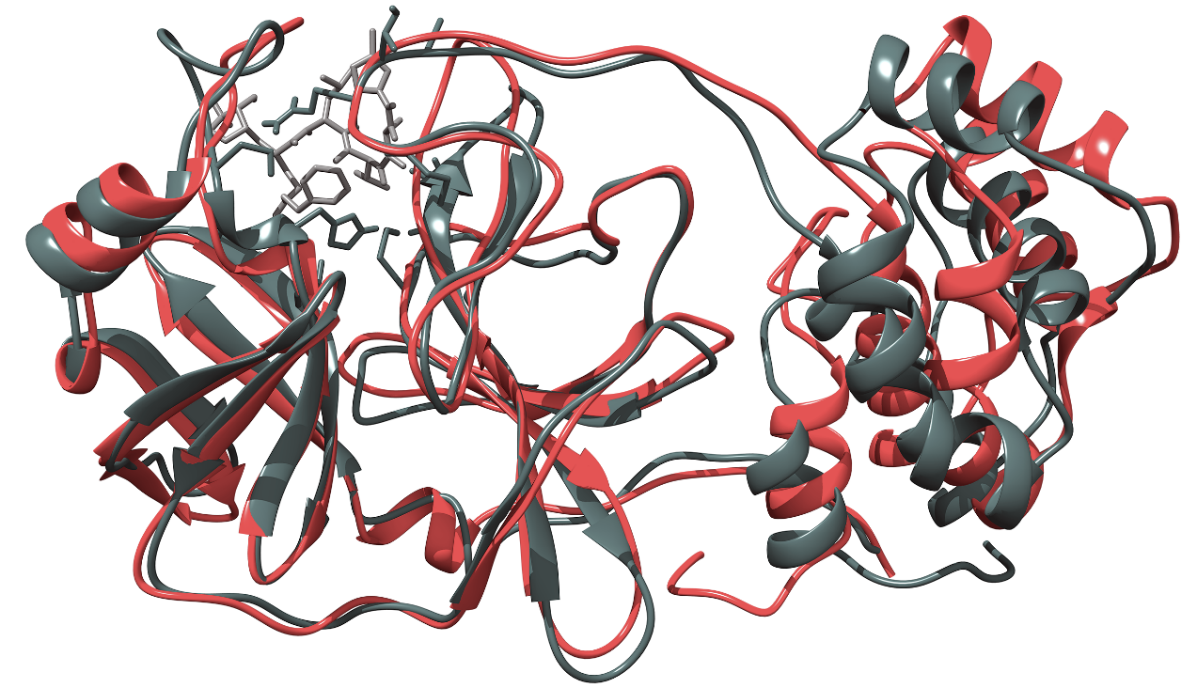


Figure S5: Average structure obtained from various conformations of MD simulation for the timescale of 1 microsecond, here the red indicates the apo protein and black indicates the protein-ligand complex


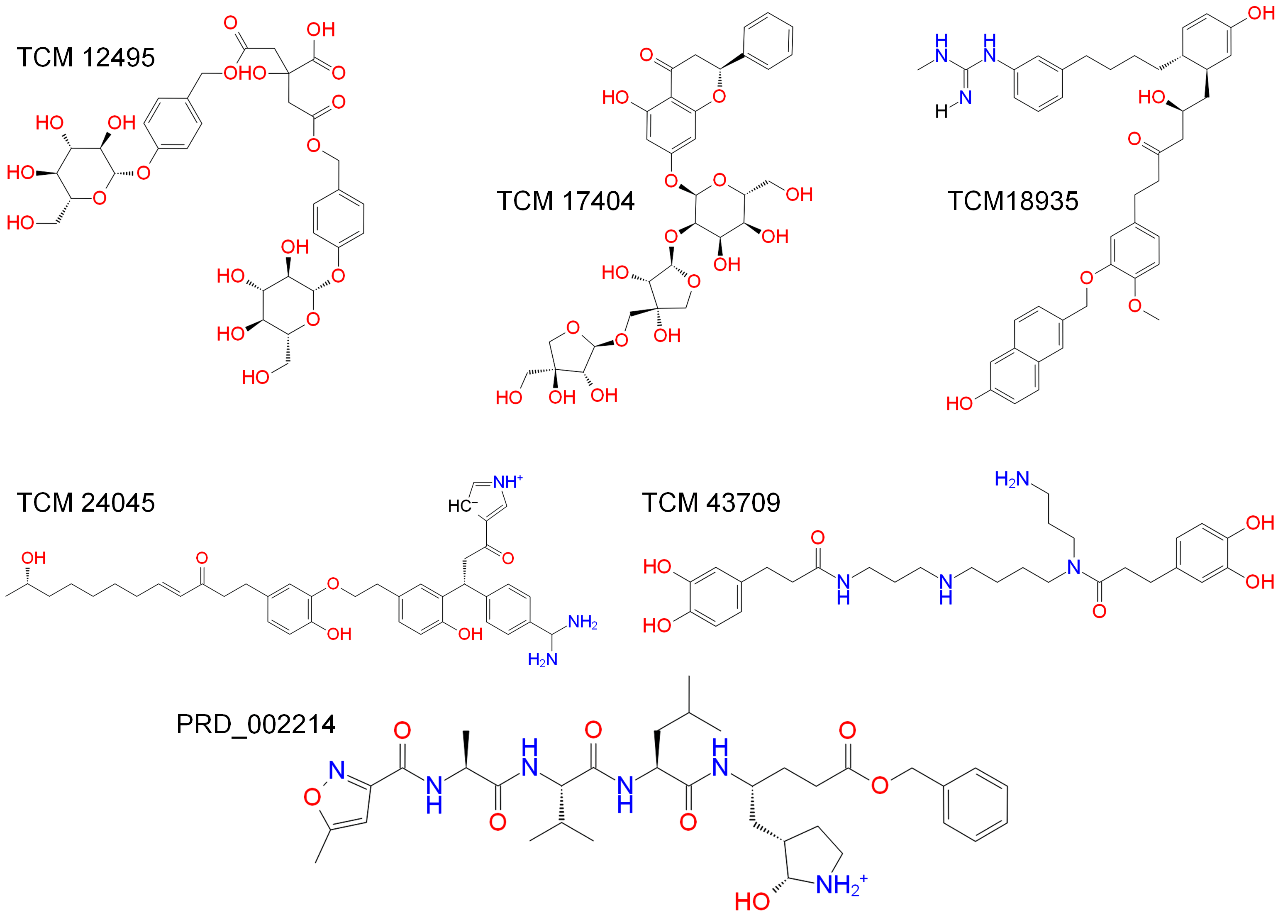


Figure S6: 2D structures of the screened compounds along with known N3 inhibitor PRD 002214 from co-crystal structure of the SARS-CoV-2 main protease (M^pro^/3CL^pro^), PDB ID: 6LU7.


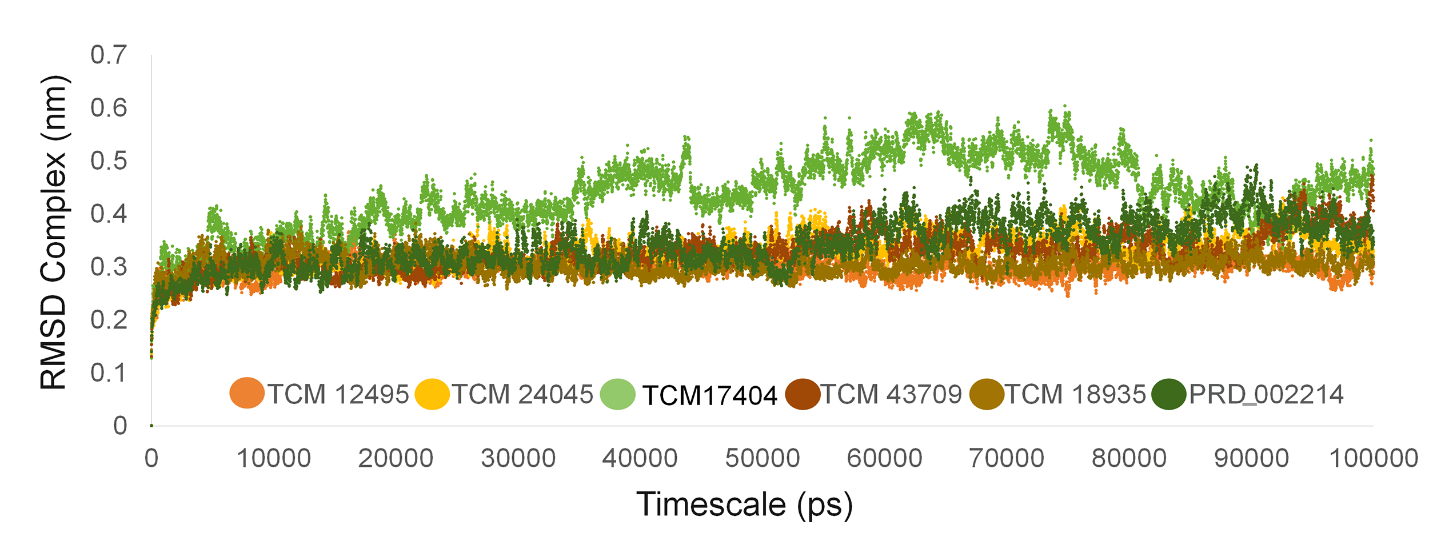


Figure S7. The RMSD values of the protein-ligand complexes over the simulation time (X-axis represents time scale in nanosecond and Y-axis represent the RMSD in nanometers).


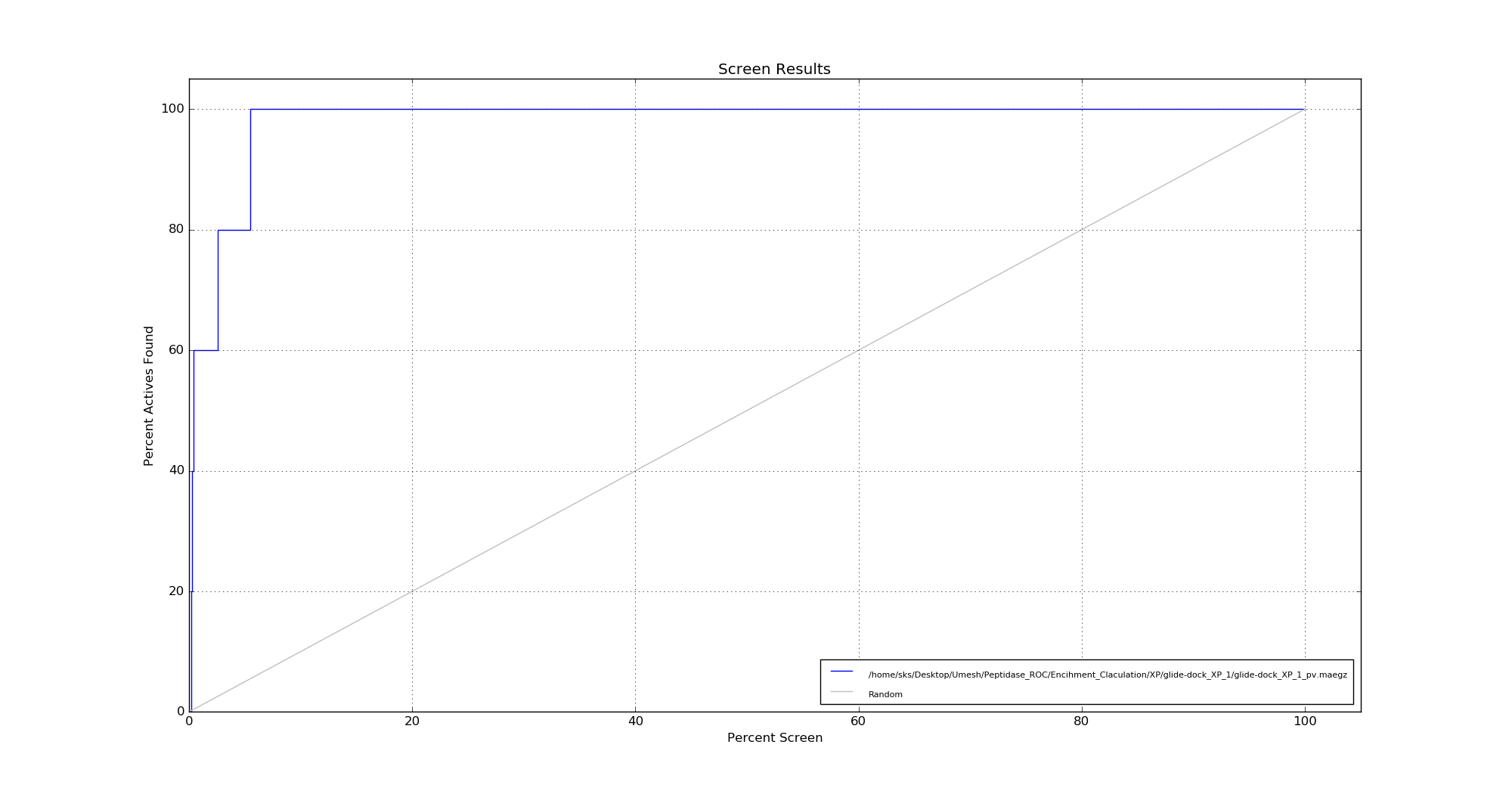


Figure S8. ROC curve for the TCM screened compounds, known compound and the random decoy set


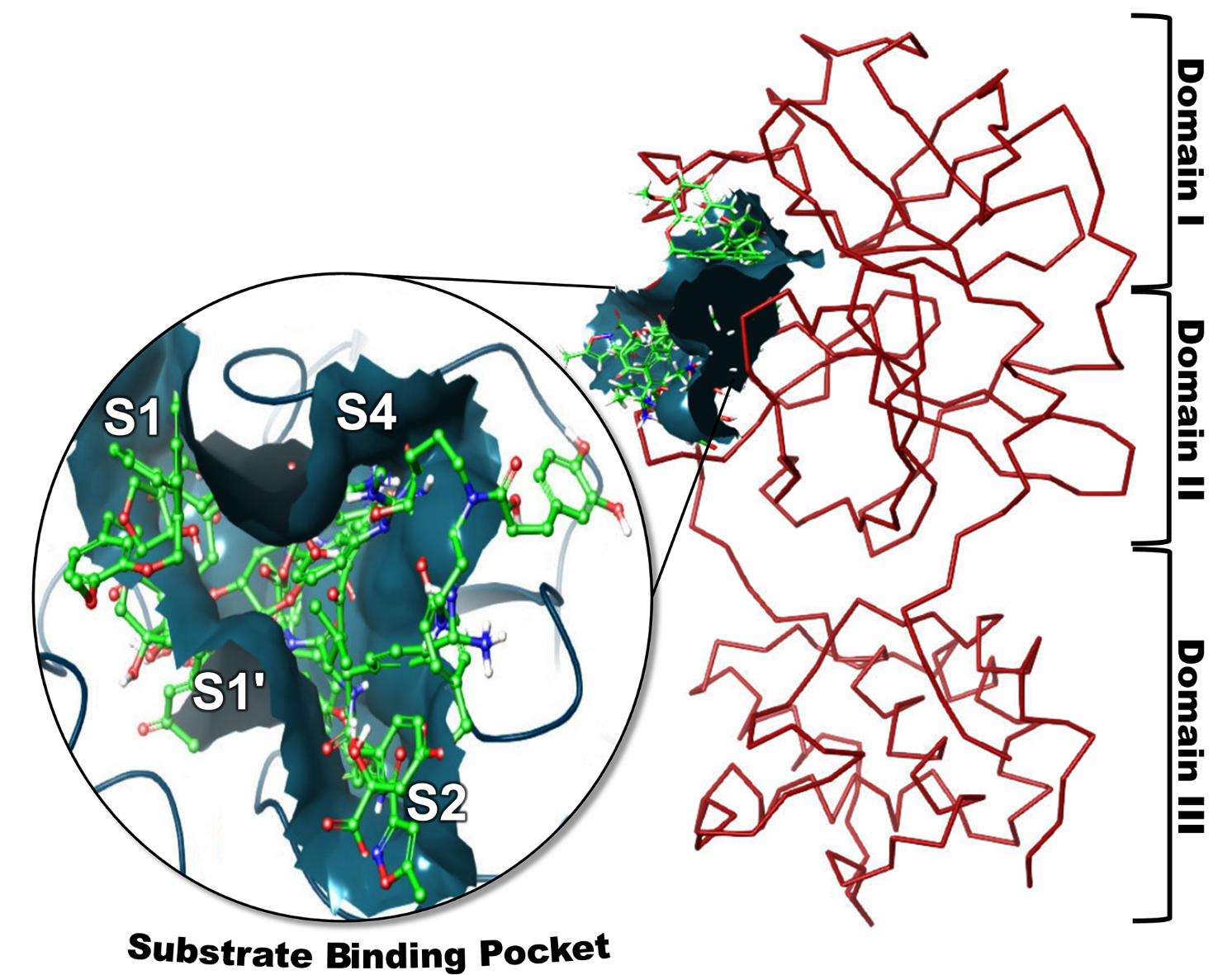


**Figure S9:** Focused view of TCM screened compounds along with co-crystal ligand compound in the binding site of SARS-CoV-2
